# Pioneering molecular screening for cervical precursor lesions and cervical cancer in sera

**DOI:** 10.3389/fonc.2024.1483882

**Published:** 2024-11-14

**Authors:** Paulina Miranda-Falconi, Gonzalo Flores-Peña, Mauro F. Jiménez-Trejo, Yazmin E. Torres-Paz, Diego O. Reyes-Hernández, Juan C. Estrada-Guzmán, Ernesto Hernández-Ramírez, Erick N. Torres-Torralba, Juan P. Rangel-Ordoñez, Daniela K. Vejar-Galicia, Patricia Reyes-Fonseca, Omar P. Islas-Bayona, Rodolfo Hernández-Paredes, Mercedes Gutiérrez, Orlando Santillán

**Affiliations:** ^1^ Women’s Medical Center, Ginequito Hospital, Monterrey, Nuevo Leon, Mexico; ^2^ Molecular Laboratory, TIMSER Group, Mexico City, Mexico; ^3^ Research Department, ATSO Pharma, Mexico City, Mexico

**Keywords:** cervical cancer screening, cervical cancer biomarker, molecular screening, cervical precursor lesions, low-grade squamous intraepithelial lesions, high-grade squamous intraepithelial lesions

## Abstract

Cervical cancer is a significant public health issue in Mexico and many developing countries. Early detection is crucial for combating this disease. The official screening test for cervical cancer is cytology, but this technique faces several barriers, including methodological, educational, and sociocultural challenges. Liquid-based cytology is an improved version of this test, however it does not address the aforementioned complications. Biomarkers for cervical precursor lesions and cervical cancer can improve timely detection of the disease. A previous study from our group identified four circulating human proteins as potential biomarkers for these conditions. For molecular screening, we selected GAPDH as the biomarker for cervical precursor lesions and HNRNPA1 as the biomarker for cervical cancer -chosen from the three previously identified options based on antibody availability- to be detected in sera. Participants underwent a comprehensive panel of tests, including liquid-based cytology, PCR detection of Human papillomavirus (HPV), colposcopy, and histopathology -when applicable-. The last two tests were used as references for determining sensitivity and specificity, with histopathology being the gold standard for cervical cancer diagnosis. All the participants successfully received colposcopies (n = 99) and only those women with visible or suspected cervical lesions/malignancies were biopsied (n = 62). A subset of randomly selected biopsies underwent p16^INK4a^ immunohistochemistry (n = 36). This study compares the performance of liquid-based cytology with the molecular screening. With colposcopy as reference, liquid-based cytology showed 30% sensitivity and 96% specificity, while the molecular screening showed 90% sensitivity and 43% specificity. With histopathology as reference, liquid-based cytology showed 21% sensitivity and 93% specificity, while the molecular screening showed 85% sensitivity and 61% specificity. The molecular screening outperformed the liquid-based cytology in several areas, including detecting true-positive cases, reducing false-negative cases by 34.62%, application time, simplicity of result´s categories, and acceptance among participants. An ideal screening test requires high sensitivity, maintains moderate specificity, and minimizes false negatives. Our proposed screening test meets these criteria, making it an ideal complement -or alternative- for cervical cancer screening.

## 1 Introduction

Cervical cancer (CC) is often asymptomatic, making early detection challenging (1). CC diagnosis is complex, particularly at the initial stages, known as cervical precursor lesions, cervical intraepithelial neoplasia (CIN-1, CIN-2, and CIN-3) or low/high-grade squamous intraepithelial lesions (LSIL or HSIL) (2, 3). The World Health Organization (WHO) has called for the development of high-performance tests for CC screening as part of its global strategy to eliminate CC as a public health problem (4).

In 2022, CC was the fourth most common cancer in incidence (662,301 new cases) and mortality (348,874 deaths) among women globally (5). In Mexico, CC was the second most common cancer, with 10,348 new cases, and 4,909 deaths in the same year (5). Due to these differences, it is likely that the frequency of the clinical stages of the disease may differ significantly between the two.

Timely detection of cervical precursor lesions and CC is difficult due to the pathology’s complexity. Cervical precursor lesions can regress to previous states (lower grade lesions or non-lesion) in some women and progress to CC in others. There is no clinical algorithm to accurately predict disease aggressiveness or its development (3). Several factors contribute to CC development, including young age at sexual debut (before 18 years or close to menarche), multiple vaginal deliveries (≥4), tobacco smoking, deficiencies in folate and vitamins (specially A, C, and E), multiple lifetime sexual partners, long-term oral contraceptive use, Human papillomavirus (HPV) infection, co-infection with other sexually transmitted pathogens, previous cervical precursor lesions (both clinically treated and untreated), and lack of regular cervical screening testing (6–19). Until today, the contribution of each independent factor is still not fully assessed, however the WHO has focused on HPV infection as the most critical etiologic factor for CC (20).

The official screening test for CC is cervical cytology in most countries in America (including Mexico), Asia, and Europe (21). However, this test has multiple barriers, such as its invasive nature, the need for specialized equipment and highly trained personnel, low sensitivity (11-57%), and a wide range of specificity (14-97%) (22–27). Additionally, cytology results heavily depend on the technical expertise of the personnel handling the samples (22). Cytology can also show 25% of false-negative rate, misclassifying 25 out of 100 women as negative when they actually have cervical precursor lesions or CC (22). These barriers prevent cytology from meeting the ideal characteristics of a screening test. Since 2021, the WHO recommends using HPV nucleic acids detection as the standard screening test instead of cervical cytology (20). However, adopting HPV detection faces obstacles, including the fact that most of HPV-infected women clear the infection within an average of 24 months; there is no cure for HPV infection; and HPV testing also requires a cervical swab, which is invasive (17, 28). The WHO recommends HPV vaccination for girls aged 9 to 14 (29). In Mexico, only 5% of the target population of girls aged 9 to 11 had completed the full vaccination scheme by 2021 (30). Mexican health authorities consider HPV detection only as a complementary test to cytology (31).

Considering the complexity of CC, molecular tests can improve cervical screening performance (32). To this end, different molecular biomarkers for CC have been studied, such as CA125, HE4, SCCA, and VEGF (33–35). Our group previously identified a set of four protein biomarkers: GAPDH for cervical precursor lesions and EIF4A1, FDPS, and HNRNPA1 for CC (36). These biomarkers were tested in a cohort of 212 Mexican women living in cities (36). Here, we propose a molecular screening test based in the immunodetection of GAPDH and HNRNPA1, the later chosen for its available antibody. This test is done with serum from a whole blood sample (obtained by venipuncture). Liquid biopsy overcomes the invasive nature of conventional cervical screening by avoiding gynecological examination (37). Molecular screening tests with high sensitivity could complement cervical cytology (37). To this end, optimization of the molecular tests is required. For this molecular screening of cervical precursor lesions and cervical cancer, optimal cutoff values for each biomarker were determined using ROC curves with histopathology as the reference. This study compares the performance of the molecular screening, liquid-based cytology, and HPV detection by PCR using colposcopy and histopathology (gold standard) as references. A total of 99 women from the general population were enrolled in the study, each receiving a comprehensive gynecological evaluation, including liquid-based cytology, the molecular screening, HPV detection by PCR, and colposcopy. In accordance with Mexican clinical guidelines and the clinical expertise of the gynecologist, only 62 women underwent biopsies (19, 31, 38). Colposcopy was used as the reference for comparing the results of all participants.

## 2 Materials and methods

### 2.1 Ethical statement

This study was approved by the Ethics Committee of the Angeles Pedregal Hospital (HAP2726, June 26, 2024).

### 2.2 Study design and setting

A cross-sectional study was conducted on a cohort of 99 women attending the private healthcare system at Ginequito Hospital (Monterrey, Nuevo Leon, Mexico) and the gynecologic healthcare campaign promoted by Nuevo Leon Ministry of Health. The sample size was calculated with the formula $n=p\left(1-p\right){\;(\frac{Z_{\propto /2}}{d})}^{2}$ ; where α = 0.05, p = 0.554 (the reported sensitivity for cytology), $Z_{\propto /2}=1.9599$ and d = 0.10 (marginal error) (23, 39). An extra 4% was added to the original sample size (n = 95) to correct for possible dropouts or losses to follow-up, giving n = 99. Women aged 18 years or older, who had begun sexual activity, had no pain or discomfort in the pelvic area, reported no abnormal vaginal bleeding or discharge, were asymptomatic for cervical cancer, and were not undergoing any cancer treatment were enrolled in the study. Women who have had cytology before and those who have never had it were included. Participants received medical care following the Mexican clinical guidelines and regulations for cervical cancer surveillance, prevention, and treatment (19, 31, 38). Diagnosis and treatment were based on colposcopy, histopathology, and the clinical expertise of the gynecologist (19, 31, 38). No patient was diagnosed or treated based on the molecular screening results. All participants received timely and adequate treatment/follow-up.

### 2.3 Mexican and international clinical guidelines

Primary prevention of cervical cancer includes providing information, counseling, and HPV vaccination to the general population (20, 31). Secondary prevention depends on the timely detection of precursor lesions and cervical cancer and its adequate treatment (20, 31, 38). These are the general recommendations of the Mexican clinical guidelines (at the first and second level of care), the Mexican Official Standard (NOM-014-SSA2-1994), and the WHO guideline for screening and treatment of cervical pre-cancer lesion for cervical cancer prevention (19, 20, 31, 38). Mexican healthcare authorities recommend liquid-based cytology for cervical cancer screening whenever possible, but the official primary screening test is conventional cytology (31). The WHO suggests using HPV DNA detection as the primary screening test, while the Mexican healthcare authorities consider it as an auxiliary test to cytology (20, 31). The Mexican clinical guideline at the first level of care states that HPV detection should be administered to women aged 30 or older, with cytology results of ASCUS or AGUS (31). This test is not indicated to women with cytology results of LSIL, HSIL, or CC because of the high prevalence of the high-risk HPV genotypes among these clinical groups (31).

Mexican clinical guidelines state that if cytology detects an abnormal result, the patient must be referred to colposcopy (31, 38). The treating gynecologist can take a biopsy if lesions or malignancies are visualized (38). Histopathologic analysis of the biopsy is considered the final diagnosis (19, 38). HPV detection can complement cytology, but it is not considered the primary screening test (31, 38).

The WHO clinical guideline considers two scenarios when the HPV DNA is detected in the primary screening test: 1) treat the patient (screen-treat approach), and 2) use partial HPV genotyping, colposcopy, cytology, or visual inspection with acetic acid to confirm diagnosis (screen-triage-treat approach). Neither approach requires confirmatory diagnosis by histopathology (20).

### 2.4 Health questionnaire

The questionnaire was part of the gynecologic health campaign promoted by the Nuevo Leon Ministry of Health and all the participants completed it (n = 99). The questionnaire collected general demographic information, clinical data, sexual health, and habits of the participants, including:

- Age
- Height
- Weight
- Tobacco smoking
- Menarche
- Age of sexual debut
- Number of lifetime sexual partners
- Year of last cytology
- Year of last colposcopy
- Contraceptive methods
- Number of vaginal deliveries, abortions, and C-sections

### 2.5 Molecular screening test

This screening test utilizes a blood sample to detect circulating protein biomarkers associated with cervical precursor lesions and cervical cancer, GAPDH and HNRNPA1, respectively (36). GAPDH is detected by ELISA and HNRNPA1 by Western blotting (36). Blood samples were collected as part of the gynecologic health campaign promoted by Nuevo Leon Ministry of Health. Blood samples were drawn by venipuncture and the screening test was applied to all participants (n = 99). This test does not require a gynecologic pelvic examination.

### 2.6 Liquid-based cytology

Liquid-based cytology (LBC), which can be performed using ThinPrep and SurePath, requires a cervical swab, also known as Pap smear (40). It has a higher positive detection rate compared to conventional cytology (31, 41, 42). LBC was selected because this type of cytology can be done with the same sample used for HPV detection by PCR, minimizing discomfort for the participant (31). Cervical swabs were collected by a licensed gynecologist. As recommended by the Mexican clinical guidelines, LBC was administered before colposcopy, for this reason the study’s staff have no *a priori* knowledge of clinical groups (control, LSIL, HSIL, or CC) among participants (31, 38). None of the participants, including the cervical precursor lesions and cervical cancer patients (identified later by colposcopy and histopathology), reported having lower back pain, abnormal vaginal bleeding or discharge, or discomfort in the genital area. All LBC analysis were performed by a licensed clinical laboratory (43) and were administered to all participants (n = 99). In the Mexican Healthcare, cytology is the primary screening test for cervical cancer, it is administered to women who: 1) have had sex, 2) have risk factors for CC, 3) are immunocompromised, 4) were exposed to diethylstilbestrol -DES- in the womb, 5) have been previously treated for CIN2/3 or CC, or 6) have never been screened (31).

### 2.7 Detection of human papillomavirus by polymerase chain reaction

Detection of human papillomavirus (HPV) by polymerase chain reaction (PCR), requiring a cervical swab, was conducted by a licensed clinical laboratory (43). Cervical swabs were collected by a licensed gynecologist. The participant’s cervical swab was used for LBC and HPV detection by PCR. This PCR test identifies 15 HPV genotypes, including:

- HPV-16.
- HPV-18.
- Pool of 13 HPV genotypes, including: HPV-31, 33, 35, 39, 45, 51, 52, 56, 58, 59, 66, 67, and 68.

Cervical swabs were collected from all participants and this test was administered to all samples (n = 99). As with LBC, no *a priori* knowledge of clinical groups (control, LSIL, HSIL, and CC) was available at the time HPV detection by PCR was done.

### 2.8 Colposcopy

Colposcopies were performed by a licensed gynecologist at the Women’s Medical Center of Ginequito Hospital in Monterrey, Nuevo Leon, Mexico (44). The gynecologist is also certified for performing this procedure by the Mexican College of Colposcopist Gynecologists. Colposcopy is performed at the second level of care within Mexico’s public healthcare system on women with abnormal cytology results (38). During this procedure, the gynecologist can take a biopsy from the patient if any cervical lesions/malignancies are visually identified (38). Colposcopy was conducted for all participants (n = 99) because it is the second-level procedure and can be used as reference for comparing screening tests (38). For participants with normal findings, colposcopy represents the final diagnosis.

### 2.9 Histopathology

Histopathologic analysis of biopsies, taken during colposcopy, is the gold standard for diagnosing cervical cancer (19). This analysis was done by a licensed clinical pathology laboratory (45). Biopsies were obtained from participants with visually identified or suspected cervical lesions and/or malignancies (n = 62) by the study gynecologist (19, 38). Six biopsies were randomly selected for a second histopathologic analysis by a different clinical pathology laboratory (46). Of these, 67% (4 out of 6) obtained a concordant result, while 33% (2 out of 6) were discordant. The final status of discordant histopathological results was determined based on colposcopic findings. None of the participants were biopsied more than once. For participants who underwent a biopsy, histopathology represents the final diagnosis.

### 2.10 p16INK4a immunohistochemistry

The p16^INK4a^ immunohistochemistry study was conducted by a licensed clinical pathology laboratory. We randomly selected 36 biopsies for p16^INK4a^ immunostaining, performed in the remaining tissue from the original biopsies. None of the participants were biopsied more than once for this test.

### 2.11 Dichotomization of test results

Categorical data of cytology, HPV detection by PCR, colposcopy, and histopathology were dichotomized (positive/negative) for comparison as follows:

**Table:** 

Molecular screening	
Positive	Negative
• GAPDH sample ≥ 17.74 ng/mL, and/or • HNRNPA1 sample ≥ 0.38 intensity units (IU)	• GAPDH sample < 17.74 ng/mL, and • HNRNPA1 sample < 0.38 IU

GAPDH, glyceraldehyde-3-phosphate dehydrogenase; HNRNPA1, heterogeneous nuclear ribonucleoprotein A1.

**Table:** 

Liquid-based cytology	
Positive	Negative
• Mild dysplasia CIN-1 • Moderate dysplasia CIN-2 • Severe dysplasia CIN-3 • Carcinoma *in situ* CIN-3 • Invasive/microinvasive cancer • Adenocarcinoma • Unspecified malignant neoplasm/malignancy • Probable CIN/dysplasia/carcinoma/adenocarcinoma/malignancy	• Negative for lesion and/or cancer • Negative with inflammation (mild, moderate, or severe) • HPV cytopathic changes • Herpes virus cytopathic changes • *Trichomonas vaginalis* (Trichomoniasis) • Bacterial vaginosis • Fungal vaginosis • Atrophy/Cellular atrophy (mild, moderate, or severe)

CIN, cervical intraepithelial neoplasia; HPV, human papillomavirus.

**Table:** 

HPV detection by PCR	
Positive	Negative
• HPV-16 detected, or • HPV-18 detected, or • HPV-pool detected	• HPV-16 not-detected, and • HPV-18 not-detected, and • HPV-pool not-detected

HPV, human papillomavirus; HPV-pool includes genotypes: 31, 33, 35, 39, 45, 51, 52, 56, 58, 59, 66, 67, and 68.

**Table:** 

Colposcopy	
Positive	Negative
• Mild dysplasia CIN-1 • Moderate dysplasia CIN-2 • Severe dysplasia CIN-3 • Carcinoma *in situ* CIN-3 • Neoplasm/invasive neoplasm • Low and High grade squamous intraepithelial lesions (LSIL and HSIL) • Probable CIN/LSIL/HSIL/lesion	• No alterations • Inflammation (mild, moderate, or severe) • HPV/condyloma/condylomatosis • Atrophy (mild, moderate, or severe) • Squamous metaplasia (mature/immature) • Cervical ectropion/glandular eversion of the cervix/cervical erosion • Naboth cysts • Cervical polyp • Lichen sclerosus

CIN, cervical intraepithelial neoplasia.

**Table:** 

Histopathology	
Positive	Negative
• Mild dysplasia CIN-1 • Moderate dysplasia CIN-2 • Severe dysplasia CIN-3 • Carcinoma *in situ* CIN-3 • Invasive/microinvasie cancer • Adenocarcinoma • Sarcoma and other tumors • Unspecified malignancy	• Normal cervical tissue • Cervicitis (acute or chronic) • Viral infection (HPV or Herpes)

CIN, cervical intraepithelial neoplasia.

Molecular screening results are categorized as either “risk” (positive) and “no-risk” (negative). A sample is considered positive if any biomarker reading exceeds its cutoff value; conversely, a result is negative only if all biomarker readings are below their respective cutoff values. Positive results are mutually exclusive for all clinical tests, except for HPV detection, where multiple genotypes can be present in the same sample. For a negative HPV detection result, none of the three target options (HPV-16, 18, or the pooled genotypes) should be detected in the sample.

### 2.12 Stratification of dichotomized test results

The dichotomized test results were stratified according to the clinical diagnosis of colposcopy and histopathology. Stratification was done according to the following rules:

**Table:** 

Colposcopic or Histopathologic diagnosis	Dichotomized test result	Group assignment
Negative	Negative	Control
Negative	Positive	Discordant
Mild dysplasia CIN-1, LSIL, or probable CIN-1/LSIL	Negative	Discordant
Mild dysplasia CIN-1, LSIL, or probable CIN-1/LSIL	Positive	LSIL
Moderate/severe dysplasia, HSIL, or carcinoma *in situ* (CIN-2/3)	Negative	Discordant
Moderate/severe dysplasia, HSIL, or carcinoma *in situ* (CIN-2/3)	Positive	HSIL
Neoplasm, cancer, invasive/microinvasive cancer, adenocarcinoma, sarcoma, unspecified malignancy	Negative	Discordant
Neoplasm, cancer, invasive/microinvasive cancer, adenocarcinoma, sarcoma, unspecified malignancy	Positive	Cervical cancer

CIN stands for cervical intraepithelial neoplasia; LSIL for low-grade squamous intraepithelial lesion; HSIL for high-grade squamous intraepithelial lesion.

### 2.13 Data parsing and processing

Data from questionnaires, molecular screening and clinical tests (LBC, colposcopy, histopathology, and immunohistochemistry p16^INK4a^) were recorded in a spreadsheet. Postal code and settlement type data was obtained from the Mexican Post Service (SEPOMEX) (47). Settlement and municipality socioeconomic data was obtained from the National Council for Evaluation of Social Development Policy (CONEVAL). The postal code reported by participant was used to determine the socioeconomic status of her settlement and municipality (48). Body mass index (BMI), age, dichotomous test results, settlement type, socioeconomic level, time since last cytology and colposcopy, and time since menarche to sexual debut were calculated using an *ad hoc* Python script. All data were verified with the original sources (**Supplementary Table S1**).

### 2.14 Statistical analyses

Statistical analysis and plots were done using R software, version 4.3.2 (Eye Holes) (49). Density plots, bar plots and pie charts were done using R package *ggplot2* (50). Contingency tables were constructed counting the number of concordant and discordant results between a test and its reference (colposcopy or histopathology). Total counts per category (true positives, true negatives, false positives, and false negatives) were recorded in the appropriate cells of the contingency tables as follows:

**Table:** 

		Reference	
		Positive	Negative
**Test**	*Positive*	true positive (TP)	false positive (FP)
	*Negative*	false negative (FN)	true negative (TN)

Sensitivity, specificity, and predictive values were calculated using the standard formulas reported in literature (51–53):


$$Sensitivity=\;\frac{TP}{TP+FN}$$

$$Specificity=\;\frac{TN}{TN+FP}$$

$$Positive\;Predictive\;Value=\;\frac{TP}{TP+FP}$$

$$Negative\;Predictive\;Value=\;\frac{TN}{TN+FN}$$

DeLong test was done with R package *pROC* (54). Cohen’s kappa test was done with R package *psych* (55). Significance level of α = 0.05 was used for all statistical tests.

## 3 Results

Participants were enrolled at Ginequito Hospital, Monterrey, Nuevo Leon, Mexico. All participants gave written informed consent, answered a questionnaire, donated a blood sample, received liquid-based cytology (LBC), human papillomavirus (HPV) detection by PCR and colposcopy tests (n=99). Biopsies were taken from participants with suspected or visual cervical lesions, in accordance with the Mexican clinical guidelines (n = 62) (31, 38). All biopsies were analyzed by histopathological studies. Six biopsies were randomly selected to be re-analyzed by a second histopathological laboratory with colposcopy results used to resolve discordant diagnosis (2/6). To complement histopathological diagnoses, 36 biopsies were randomly selected for p16^INK4a^ immunohistochemistry studies. None of the participants were biopsied more than once. Colposcopy (n = 99) and histopathology (n = 62) were used as references for test comparisons.

### 3.1 Demographics

Demographics, sexual health, and reproductive information were collected from participants using a questionnaire (**Table 1**). Response rates varied from 42% to 100%, with the lowest being the *year of previous colposcopy* question (57 participants did not answer). For questions regarding the year of previous cytology, both conventional and liquid-based, 13 participants did not answer and 8 responded they have never received the test before the present study.

**Table 1:** General demographics, sexual, and reproductive information.

Variable	n	Mean	SEM	Min	Max	Mode
Age	99	42.96	1.10	20	75	47
BMI	94 ^a^	29.73	0.63	19.47	50.78	24.80
Age at menarche	99	12.37	0.17	8	16	12
Age at sexual debut	99	19.82	0.49	13	38	18
Number of years since menarche to sexual debut	99	7.44	0.49	1	27	6
Number of lifetime sexual partners	97 ^a^	2.33	0.15	1	≥4	1
Number of years since last cytology	86 ^b^	2.64	0.39	0 ^c^	21 ^d^	1
Number of years since last colposcopy	42 ^e^	3.13	0.82	0 ^c^	11 ^f^	4
Number of abortions	94 ^a^	0.32	0.06	0	3	0
Number of vaginal deliveries	83 ^a^	0.94	0.14	0	5	0
Number of C-sections	83 ^a^	1.34	0.14	0	5	0
Number of cigarettes per week	99 ^g^	21.87	6.80	1	140	8

BMI, body mass index; Max, maximum; Min, minimum; SEM, standard error of the mean. **^a^** Some of the questions were not answered by all the 99 participants. **^b^** 8 participants responded they have never received cytology and 13 did not answer. **^c^** A 0 value means the patient received a previous cytology or colposcopy during the same year of her participation in the present study. **^d^** Given 8 participants responded they have never received a cytology, the maximum number could be in the interval 5 – 45 years, i.e, the age of these participants minus their age at sexual debut. These data were not included in the table because they are estimates. **^e^** 26 participants responded they have never received a colposcopy and 57 did not answer. **^f^** Given 26 participants responded they have never received a colposcopy, the maximum number could range from 5 – 45 years, i.e., the age of these participants minus their age at sexual debut. These data were not included in the table because they are estimates. **^g^** All participants answered this question, but only 23 participants responded they have smoked at some point in their lifetime (including quitters and persistent smokers). Central tendency measures were calculated based on the 23 smokers.

As seen in **Table 1**, participants averaged 43 years old, had a BMI of approximately 29.7, menarche at 12 years, had their first sexual intercourse at 20 years, had two lifetime sexual partners, received her previous cytology and colposcopy around 3 years prior, had one vaginal delivery and one C-section, no abortions, and had never smoked tobacco cigarettes. Data distribution per demographic variable is shown in **Supplementary Figures S1–S13**.

Socioeconomic data showed that 12.12% (12 out of 99) of participants resided in rural areas, while the remaining 87.88% (87 out of 99) inhabited urban areas (**Supplementary Figure S14**). Municipal poverty level among participants (**Supplementary Figure S15**) was:

- Medium 5.10% (5/99),
- Low 12.12% (12/99), and
- Very-low 82.83% (82/99).

### 3.2 Dichotomized test results

Dichotomized results were used to ease tests comparisons (**Table 2**).

**Table 2:** Dichotomized tests results.

Test	n	Results	
		Positives	Negatives
LBC	99	12	87
Molecular screening	99	66	33
HPV detection by PCR	99	9	90
Colposcopy	99	30	69
Histopathology	62	34	28
Immunohistochemistry p16^INK4a^ ^*^	36	4	26

Not all participants were biopsied. None of the participants was biopsied more than once. The p16^INK4a^ immunostaining was done in 36 randomly selected biopsies. Abbreviations: HPV, human papillomavirus; LBC, liquid-based cytology; PCR, polymerase chain reaction.

^*^ Immunohistochemistry p16^INK4a^ reported 6 biopsies as *insufficient for diagnosis*.

As seen in **Table 2**, LBC and HPV detection by PCR reported lower positive-results, 12% (12 out of 99) and 9% (9 out of 99), respectively. Colposcopy showed 30% (30 out of 99) of positive results ratio. These findings suggest a bias towards negative results for these tests. Molecular screening and histopathology reported higher positive-results ratios, 67% (66 out of 99) and 55% (34 out of 62), respectively. The molecular screening showed a bias towards positive results.

As previously mentioned, the Mexican clinical guidelines specify that biopsies should only be taken from women with suspected and/or visually identified cervical lesions or malignancies during a colposcopic examination (38). In the present study, only 62 women were biopsied. For these clinical reasons, we used colposcopy (n = 99) and histopathology (n = 62) as independent references for comparing LBC, molecular screening, and HPV detection by PCR.

### 3.3 Contingency tables

Based on dichotomized test results, we determined the number of concordant and discordant outcomes between the tests and both references (**Tables 3**–**8**). Additionally, we compared the results between colposcopy and histopathology, with the later serving as the gold standard (**Table 9**).

**Table 3:** LBC vs colposcopy.

		Colposcopy	
		Positive	Negative
LBC	Positive	9	3
	Negative	21	66

LBC, liquid-based cytology.

**Table 4:** Molecular screening vs colposcopy.

		Colposcopy	
		Positive	Negative
Molecular screening	Positive	27	39
	Negative	3	30

Molecular screening of cervical precursor lesions and cervical cancer.

**Table 5:** HPV detection by PCR vs colposcopy.

		Colposcopy	
		Positive	Negative
HPV detection by PCR	Positive	3	6
	Negative	27	63

HPV, human papillomavirus; PCR, polymerase chain reaction.

**Table 6:** LBC vs histopathology.

		Histopathology	
		Positive	Negative
LBC	Positive	7	2
	Negative	27	26

LBC, liquid-based cytology.

**Table 7:** Molecular screening vs histopathology.

		Histopathology	
		Positive	Negative
Molecular screening	Positive	29	11
	Negative	5	17

Molecular screening of cervical precursor lesions and cervical cancer.

**Table 8:** HPV detection by PCR vs histopathology.

		Histopathology	
		Positive	Negative
HPV by PCR	Positive	4	1
	Negative	30	27

HPV, human papillomavirus; PCR, polymerase chain reaction.

**Table 9:** Colposcopy vs histopathology.

		Histopathology	
		Positive	Negative
Colposcopy	Positive	23	7
	Negative	11	21

Histopathology is used as reference.

### 3.4 Sensitivity, specificity, and predictive values

Sensitivity, specificity, and predictive values for each test used histopathology (**Table 10**) and colposcopy (**Table 11**) as references.

**Table 10:** Sensitivity, specificity, and predictive values using histopathology as reference.

Test	Sensitivity (%)	Specificity (%)	PPV (%)	NPV (%)
LBC	20.59	92.86	77.78	49.06
Molecular screening	85.29	60.71	72.50	77.27
HPV detection by PCR	11.76	96.43	80.00	47.37
Colposcopy	67.55	75.00	76.67	65.63

LBC, liquid-based cytology; HPV, human papillomavirus; PCR, polymerase chain reaction; NPV, negative predictive value; PPV, positive predictive value. These values were calculated using standard formulas (51–53). Only 62 participants were biopsied.

**Table 11:** Sensitivity, specificity, and predictive values using colposcopy as reference.

Test	Sensitivity (%)	Specificity (%)	PPV (%)	NPV (%)
LBC	30.00	95.65	75.00	75.86
Molecular screening	90.00	43.48	40.91	90.91
HPV detection by PCR	10.00	91.30	33.33	70.00

HPV, human papillomavirus; LBC, liquid-based cytology; PPV, positive predictive value; NPV, negative predictive value; PCR, polymerase chain reaction. Standard formulas were used to calculate these values (51–53). All participants received colposcopies (n = 99).

As seen in **Table 10**, the molecular screening outperformed LBC by 64.70% in sensitivity, demonstrating a better ability to correctly identify participants with a pathological state (cervical precursor lesions and CC). However, the molecular screening was 30.15% less specific than LBC, identifying fewer healthy women as negatives. In cancer screening programs, it is critical to detect individuals with the disease promptly; therefore, sensitivity is one of the most important parameters. The area under the ROC curve of the molecular screening was significantly different from that of LBC (DeLong test, *P-value* < 0.05). The agreement between the molecular screening and histopathology was moderate (Cohen’s kappa = 0.47, 95% confidence interval: 0.25 – 0.69), while that of LBC and histopathology was slight (Cohen’s kappa = 0.12, 95% CI: -0.032 – 0.28), according to the Landis-Koch scale (56).

To strengthen histopathology diagnosis, 36 randomly selected biopsies (36 out of 62) were tested for immunohistochemistry p16^INK4a^. The human protein p16^INK4a^ is overexpressed in HPV infected cells and is used as a biomarker for this viral infection (57–60). p16^INK4a^ immunostaining results were as follows: 6 samples (16.67%) were insufficient for diagnosis, 26 (72.22%) were negative, and 4 (11.11%) were positive. Of these, there were 3 true positives, 1 false negative, 10 false positives, and 16 true negatives, using histopathology as the reference. The sensitivity of immunohistochemistry p16^INK4a^ was 75.00%, specificity was 61.54%, positive predictive value was 23.08%, and negative predictive value was 94.12% (reference: histopathology). The agreement between p16^INK4a^ and histopathology was slight (Cohen’s kappa = 0.19, 95% CI: -0.09 – 0.46) according to the Landis-Koch scale (56).

To analyze all participants’ data (n = 99), we used colposcopy as reference (**Table 11**).

### 3.5 Clinical group assignment based on colposcopy and histopathology

The stratified dichotomic test results allowed us to classify them as pathologic status (low-grade squamous intraepithelial lesions, LSIL; high-grade squamous intraepithelial lesions, HSIL; and cervical cancer, CC) and controls. As seen in contingency tables, some of the tests’ results were discordant with one or both references used in this study, which are classified as false positives and false negatives (**Table 12**).

**Table 12:** Stratified dichotomous tests results using colposcopy and histopathology as references.

Group	Diagnosis		Colposcopy as reference			Histopathology as reference			
	Colp	Histopat	LBC	Mol	HPV	LBC	Mol	HPV	Colp
Control	69	28	66	30	63	26	17	27	21
LSIL	25	31	6	24	3	7	27	3	21
HSIL	4	2	3	2	0	0	1	1	1
CC	1	1	0	1	0	0	1	0	1
Discordant	N/A	N/A	24	42	33	29	16	31	18
**Total**	**99**	**62**	**99**	**99**	**99**	**62**	**62**	**62**	**62**

CC, cervical cancer; Colp, colposcopy; Histopat, histopathology; HSIL, high-grade squamous intraepithelial lesions; LBC, liquid-based cytology; LSIL, low-grade squamous intraepithelial lesions; Mol, molecular screening; N/A, not applicable; HPV, human papillomavirus detection by PCR. Total number of participants per clinical test are shown in bold.

As seen in **Table 12**, using histopathology as the reference, molecular screening identified 85.29% (29 out of 34) of the women with any pathological status (cervical precursor lesions and CC) while LBC only identified 20.59% (7 out of 34) of these women. When using colposcopy as the reference, molecular screening identified 90% (27 out of 30) and LBC identified 30% (9 out of 30) of these women.

### 3.6 Features of an ideal screening test

Using both colposcopy and histopathology as references, we compared the results according to the features of an ideal screening test (**Table 13**) (61).

**Table 13:** Comparison of results -using colposcopy and histopathology as references- to an ideal screening test.

Ideal screening test (features)	Colposcopy			Histopathology		
	LBC	Mol	HPV	LBC	Mol	HPV
High sensitivity	30.00%	90.00%	7.41%	20.59%	85.29%	11.76%
False Negatives (low proportion)	70.00% (21/30)	10.00% (3/30)	90.00% (27/30)	79.41% (27/34)	14.71% (5/34)	88.24% (30/34)
False Positives (high proportion)	4.35% (3/69)	56.52% (39/69)	8.70% (6/69)	7.14% (2/28)	39.29% (11/28)	3.57% (1/28)
Quick implementation	30-60 min	4-6 min	30-60 min	30-60 min	4-6 min	30-60 min
Simple (design & analysis)	Visual R: 9 categories	Molecular R: risk/no risk	Molecular R: Detected (genotype)/Not detected	Visual R: 9 categories	Molecular R: risk/no risk	Molecular R: Detected (genotype)/Not detected
High acceptance level	Medium-low ^1^	Good ^2^	Medium-low ^1^	Medium-low ^1^	Good ^2^	Medium-low ^1^
Minimal disturbance	Cervical swab	Venipuncture	Cervical swab	Cervical swab	Venipuncture	Cervical swab
Affordable cost	$64.60 USD	$43.93 USD	$64.60 USD	$64.60 USD	$43.93 USD	$64.60 USD

LBC, liquid-based cytology; Mol, molecular screening; HPV, human papillomavirus detection by PCR; R, results. *Cervical swab*: this sample was obtained during gynecological examination. *Venipuncture*: blood samples were obtained by a trained phlebotomist. Acceptance levels were assigned according to the sampling technique. LBC and HPV costs were obtained directly from final quotations. The molecular screening test cost was estimated based on the required molecular reagents, equipment, and personnel. All costs were calculated based on average official exchange rate, $19.3505 MXN (Mexican Official Journal of the Federation).

**^1^** Given both the cytology and HPV detection by PCR require the same sample type, acceptance levels for both were estimated based on the percentage of the target population that received a cytology during 2020 in Mexico (31.4%) (68).

**^2^** The molecular screening requires a blood sample. The acceptance level of phlebotomy was estimated based on the popularity of laboratory blood tests. Phlebotomy is considered as a minimum risk procedure by Mexican Health Ministry (69).

As seen in **Table 13**, molecular screening outperformed both LBC and HPV detection by PCR in each feature, regardless of the reference used for comparisons.

## 4 Discussion

Conventional cytology is the official screening test for cervical precursor lesions and cervical cancer (CC) in Mexico, but it faces multiple barriers, notably its low sensitivity, which ranges from 11–57% (22–27). Liquid-based cytology (LBC) is an improved version of conventional cytology with higher sensitivity (31, 41, 42). Sensitivity measures screening tests’ ability to correctly identify true positive cases, i.e., classifying diseased individuals as positive. After a positive cytology result, women receive colposcopy, during it the gynecologist may take a biopsy when epithelial lesions or malignancies are detected. Disease diagnosis is confirmed with the gold standard, such as histopathology (biopsy analysis). Histopathologic analyses are done exclusively on women obtaining positive results in cytology and colposcopy.

Liquid biopsy offers an alternative way of screening CC, such as detecting circulating DNA (from human and HPV origin), RNA (coding and non-coding), epigenetic modifications (methylation), or protein biomarkers (36, 37, 62–65). For example, sequences of HPV DNA (e.g., E7 and L1), methylation of cell-free DNA of MAL and CADM1 genes, and expression levels of SCCA protein, miRNA-29a, miRNA-25, and miRNA-486-5p have been detected in human sera (62–65).

An ideal screening test should have high sensitivity, allows a high proportion of false positives (healthy individuals identified as positives), and a low proportion of false negatives (diseased individuals identified as negatives). It should be simple (in design and analysis), quickly executed, well accepted by target population, minimally discomforting to patients, and as affordable as possible (61). Our results demonstrate that the molecular screening outperformed liquid-based cytology in terms of sensitivity, reducing the proportion of false-negative cases, lowering application time, reaching higher test acceptance, and a lower estimated cost. This molecular screening on serum samples detects circulating protein biomarkers associated with cervical precursor lesions (GAPDH) and CC (HNRNPA1). GAPDH participates in glycolysis, associated with the Warburg effect observed in some cancer cells (66). HNRNPA1 promotes alternative splicing of some mRNA’s oncogenes (67). The molecular screening represents an alternative for women who want a less invasive test. However, further experimentation is needed to assess the suitability of the molecular test for screening broader and more diverse populations (66, 67).

The molecular screening identified 22 true positives cases missed by LBC, including 20 cases of LSIL, one case of HSIL, and one case of CC (adenocarcinoma). The last two are the most concerning cases because if left unattended, the lives of these women would be endangered. Mexican clinical guidelines state that all women receiving two consecutive negative cytology results will be screened again in three years. Within this time frame, these women’s health could be significantly compromised. For the women in this study, the mean number of years since their last cytology was 2.64 ± 0.39 years, almost 3 years. Of the eight participants who had never received a cytology before this study, five were diagnosed with LSIL and one with HSIL by histopathology. The remaining two participants were not biopsied because they received a normal result in colposcopy.

Colposcopy was even less frequently performed, with an average of 3.13 ± 0.82 years since the last colposcopy among the women of this study. Of the 26 participants who had never received a colposcopy before, six were diagnosed as negative for cervical precursor lesion/malignancy, 11 were diagnosed with LSIL, and 2 were diagnosed with HSIL by histopathology. The remaining 7 participants were not biopsied because they received a normal result in colposcopy.

Based on these results, we believe that adopting molecular screening can offer a significant advantage in cervical cancer prevention. Timely detection of cervical precursor lesions and early stages of CC is crucial for effective intervention. Mexican clinical guidelines currently limit colposcopy to women with positive cytology results, which means many potential cases could go undetected.

Our data reveals that liquid-based cytology classified 87.88% (87 out of 99) of the participants as negative, with a concerning 31.03% (27 out of 87) of these being false negatives. In contrast, molecular screening identified only 33.33% (33 out of 99) of participants as negative, with a much lower false negative rate of 15.15% (5 out of 33). This demonstrate that molecular screening was more effective in detecting true positive cases and reduced the likelihood of false negatives. The use of more than one biomarker might explain this high accuracy, as reported by authors detecting protein and microRNA markers related to CC with 88.6% sensitivity and 92.9% specificity (65).

By integrating molecular screening into routine practice, early detection may be enhanced, the risk of missing critical cases may be reduced, and the outcomes for women will ultimately improve. Given its performance, we believe molecular screening should be considered for broader implementation in clinical settings. However, its implementation will require equipment (e.g., electrophoresis chambers, membrane transfer and imaging systems, shakers, and ELISA readers) and training for laboratory staff. These requirements may not be economically feasible for most healthcare institutions, e.g., in Mexico, conventional cytology remains the official screening test due to budget limitations (31). Developing a multiplexed ELISA kit or a rapid test -like a lateral flow assay, LFA- can bypass most of the implementation barriers. Other groups have developed LFA to detect CC biomarkers like SCCA and CA125 (33).

HPV detection by PCR obtained the lowest sensitivity values among the evaluated tests, regardless of the reference used for comparisons, with 7.41% and 11.76%, for colposcopy and histopathology, respectively. Our results support the decision of the Mexican Health Ministry to use the HPV detection test as a complement to the conventional cytology. Although the WHO recommends substituting cytology with HPV detection as the official screening test for cervical precursor lesions and CC, we believe this will not be feasible in countries like Mexico.

This study has some limitations, including lower representation of women from rural areas, and varying socioeconomic backgrounds. Additionally, the selection of two biomarkers, the sample size, and potential ethnicity bias given the focus on women from Monterrey, Nuevo Leon, Mexico.

## 5 Perspectives

Including a second CC biomarker in the molecular screening test will help us determine if its performance can be improved. Also, developing a multiplexed laboratory test like an ELISA or a rapid test like a lateral flow assay can improve this molecular screening. We believe the incorporation of the molecular screening into the Mexican healthcare system could benefit a broader female population, by correctly identifying women with cervical precursor lesions and CC.

## Data Availability

The original contributions presented in the study are included in the article/**Supplementary Material**. Further inquiries can be directed to the corresponding author.

## References

[1] Cohen PA Jhingran A Oaknin A Denny L. Cervical cancer. Lancet. (2019) 393:169–82. doi: 10.1016/S0140-6736(18)32470-X 30638582

[2] Buckley C Butler E Fox H. Cervical intraepithelial neoplasia. J Clin Pathol. (1982) 35:1–13. doi: 10.1136/jcp.35.1.1 7037858 PMC497441

[3] Sellors JW Sankaranarayanan R. Colposcopy and treatment of cervical intraepithelial neoplasia: a beginners manual. Centro Internacional de Investigaciones sobre el Cáncer (IARC, Lyon, Francia (2003). p. 1–140. Available at: https://screening.iarc.fr/colpochap.php?lang=3&chap=2 (Accessed June 29, 2024).

[4] World Health Organization. Global strategy to accelerate the elimination of cervical cancer as a public health problem (2020). Available online at: https://www.who.int/publications/i/item/9789240014107 (Accessed June 29, 2024).

[5] World Health Organization International Agency for Research on Cancer. Global Cancer Observatory (2024). Available online at: https://gco.iarc.fr/today/home Accesed: July 9, 2024).

[6] Johnson CA James D Marzan A Armaos M. Cervical cancer: an overview of pathophysiology and management. Semin Oncol Nurs. (2019) 35:166–74. doi: 10.1016/j.soncn.2019.02.003 30878194

[7] Ruiz ÁM Ruiz JE Gavilanes AV Eriksson T Lehtinen M Pérez G. Proximity of first sexual intercourse to menarche and risk of high-grade cervical disease. J Infect Dis. (2012) 206:1887–96. doi: 10.1093/infdis/jis612 23066159

[8] Hwang LY Ma Y Benningfield SM Clayton L Hanson EN Jay J. Factors that influence the rate of epithelial maturation in the cervix in healthy young women. J Adolesc Health. (2009) 44:103–10. doi: 10.1016/j.jadohealth.2008.10.006 PMC2662755 19167657

[9] McGraw SL Ferrante JM. Update on prevention and screening of cervical cancer. World J Clin Oncol. (2014) 5:744–52. doi: 10.5306/wjco.v5.i4.744 PMC4129537 25302174

[10] Bezabih M Tessema F Sengi H Deribew A. Risk factors associated with invasive cervical carcinoma among women attending Jimma University specialized hospital, southwest Ethiopia: A case control study. Ethiop J Health Sci. (2015) 25:345–52. doi: 10.4314/ejhs.v25i4.8 PMC4762973 26949299

[11] Tekalegn Y Sahiledengle B Woldeyohannes D Atlaw D Degno S Desta F. High parity is associated with increased risk of cervical cancer: Systematic review and meta-analysis of case–control studies. In: Women’s Health, vol. 18. SAGE Publications Ltd (2022). doi: 10.1177/17455065221075904 PMC8819811 35114865

[12] Collins S Rollason TP Young LS Woodman CBJ. Cigarette smoking is an independent risk factor for cervical intraepithelial neoplasia in young women: A longitudinal study. Eur J Cancer. (2010) 46:405–11. doi: 10.1016/j.ejca.2009.09.015 PMC2808403 19819687

[13] Plummer M Herrero R Franceschi S Meijer CJLM Snijders P Bosch FX. Smoking and cervical cancer: pooled analysis of the IARC multi-centric case-control study. Cancer Causes Control. (2003) 14:805–14. doi: 10.1023/B:CACO.0000003811.98261.3e 14682438

[14] Berrington De González A Green J. Comparison of risk factors for invasive squamous cell carcinoma and adenocarcinoma of the cervix: Collaborative reanalysis of individual data on 8,097 women with squamous cell carcinoma and 1,374 women with adenocarcinoma from 12 epidemiological studies. Int J Cancer. (2007) 120:885–91. doi: 10.1002/ijc.v120:4 17131323

[15] Appleby P Beral V De González AB Colin D Franceschi S Green J. Cervical carcinoma and sexual behavior: Collaborative reanalysis of individual data on 15,461 women with cervical carcinoma and 29,164 women without cervical carcinoma from 21 epidemiological studies. Cancer Epidemiol Biomarkers Prev. (2009) 18:1060–9. doi: 10.1158/1055-9965.EPI-08-1186 19336546

[16] International Collaboration of Epidemiological Studies of Cervical Cancer. Cervical cancer and hormonal contraceptives: collaborative reanalysis of individual data for 16 573 women with cervical cancer and 35 509 women without cervical cancer from 24 epidemiological studies. Lancet. (2007) 370:1609–21. www.thelancet.com. 10.1016/S0140-6736(07)61684-5 17993361

[17] Vesco KK Whitlock EP Eder M Burda BU Senger CA Lutz K. Risk Factors and Other Epidemiologic Considerations for Cervical Cancer Screening: A Narrative Review for the U.S. Preventive Services Task Force (2011). Available online at: www.annals.org (Accessed June 29, 2024). 10.7326/0003-4819-155-10-201111150-00377 22006929

[18] Ribeiro AA Costa MC Alves RRF Villa LL Saddi VA Carneiro MADS. HPV infection and cervical neoplasia: Associated risk factors. Infect Agent Cancer. (2015) 10(16):1–7. doi: 10.1186/s13027-015-0011-3 PMC4524198 26244052

[19] Mexico’s Ministry of Health. NOM-014-SSA2-1994. For prevention, detection, diagnosis, treatment, control and epidemiological surveillance of cervical cancer(2007). Available online at: https://www.gob.mx/cms/uploads/attachment/file/10397/NOM-014-SSA2-1994.pdf (Accessed May 14, 2024).

[20] World Health Organization. WHO guideline for screening and treatment of cervical pre-cancer lesions for cervical cancer prevention, 2nd ed. (2021). Geneva: World Health Organization pp. 1–115. 34314129

[21] Zhang L Mosquera I Lucas E Rol ML Carvalho AL Basu P. CanScreen5, a global repository for breast, cervical and colorectal cancer screening programs. Nat Med. (2023) 29:1135–45. doi: 10.1038/s41591-023-02315-6 PMC10202799 37106168

[22] Kitchener HC Castle PE Cox JT. Chapter 7: Achievements and limitations of cervical cytology screening. Vaccine. (2006) 24(S3):S3/63–S3/70. 10.1016/j.vaccine.2006.05.113 16950019

[23] Mayrand MH Duarte-Franco E Rodrigues I Walter SD Hanley J Ferenczy A. Human Papillomavirus DNA versus Papanicolaou Screening Tests for Cervical Cancer. New Engl J Med. (2007) 357(16):1579–88. www.nejm.org. 10.1056/NEJMoa071430 17942871

[24] Sadat Najib F Hashemi M Shiravani Z Poordast T Sharifi S Askary E. Diagnostic accuracy of cervical pap smear and colposcopy in detecting premalignant and Malignant lesions of cervix. Indian J Surg Oncol. (2020) 11:453–8. doi: 10.1007/s13193-020-01118-2 PMC7501362 33013127

[25] Fahey MT Irwig L Macaskill P. Meta-analysis of pap test accuracy. Am J Epidemiol. (1995) 141:680–9. doi: 10.1093/oxfordjournals.aje.a117485 7702044

[26] Nanda K Mccrory DC Myers ER Bastian LA Hasselblad V Hickey JD. Accuracy of the papanicolaou test in screening for and follow-up of cervical cytologic abnormalities: A systematic review. Ann Intern Med. (2000) 132:810–9. http://annals.org/. 10.7326/0003-4819-132-10-200005160-00009 10819705

[27] Barut MU Kale A Kuyumcuoğlu U Bozkurt M Ağaçayak E Özekinci S. Analysis of sensitivity, specificity, and positive and negative predictive values of smear and colposcopy in diagnosis of premalignant and Malignant cervical lesions. Med Sci Monitor. (2015) 21:3860–7. doi: 10.12659/MSM.895227 PMC4678924 26655816

[28] Centers for Disease Control and Prevention United States Government. Human Papillomavirus (HPV) Treatment and Care (2021). Available online at: https://www.cdc.gov/std/hpv/treatment.htm (Accessed June 29, 2024).

[29] World Health Organization. Human papillomavirus vaccines: WHO position paper (2022 update) (2022). Available online at: https://iris.who.int/bitstream/handle/10665/365350/WER9750-eng-fre.pdf?sequence=1 (Accessed June 29, 2024).

[30] World Health Organization. Mexico. Cervical cancer country profile (2021). Available online at: https://cdn.who.int/media/docs/default-source/country-profiles/cervical-cancer/cervical-cancer-mex-2021-country-profile-es.pdf?sfvrsn=8a0b4124_38&download=true (Accessed June 29, 2024).

[31] Mexican Social Security Institute (IMSS) Mexican Government. Clinical practice guidelines. Prevention and timely detection of cervical cancer at the first-level of care (2011). Available online at: https://www.imss.gob.mx/sites/all/statics/guiasclinicas/146GER.pdf (Accessed June 29, 2024).

[32] Ruiz Esparza Garrido R Gutiérrez M Velázquez Flores MÁ. Circulating cervical cancer biomarkers potentially useful in medical attention (Review). Mol Clin Oncol. (2023) 18(13):1–15. doi: 10.3892/mco.2023.2609 PMC9892968 36761385

[33] Xia J Liu Y Ran M Lu W Bi L Wang Q. The simultaneous detection of the squamous cell carcinoma antigen and cancer antigen 125 in the cervical cancer serum using nano-Ag polydopamine nanospheres in an SERS-based lateral flow immunoassay. RSC Adv. (2020) 10:29156–70. doi: 10.1039/D0RA05207H PMC9055935 35521095

[34] Hwang WY Suh DH Kim K Kim YB No JH. Serum human epididymis protein 4 as a prognostic marker in cervical cancer. Cancer Control. (2022) 29:1–9. doi: 10.1177/10732748221097778 PMC9072869 35506739

[35] Zusterzeel PLM Span PN Dijksterhuis MGK Thomas CMG Sweep FCGJ Massuger LFAG. Serum vascular endothelial growth factor: A prognostic factor in cervical cancer. J Cancer Res Clin Oncol. (2009) 135:283–90. doi: 10.1007/s00432-008-0442-y PMC12160239 18626660

[36] Reyes-Hernández DO Estrada-Guzmán JC Gutiérrez M Kaeller J Meza-Sánchez DE Torres-Paz YE. Novel serum protein biomarkers for precancerous cervical lesions and cervical cancer. Glob J Health Sci. (2024) 16:44. https://ccsenet.org/journal/index.php/gjhs/article/view/0/50473.

[37] Cafforio P Palmirotta R Lovero D Cicinelli E Cormio G Silvestris E. Liquid biopsy in cervical cancer: Hopes and pitfalls. Cancers (Basel). (2021) 13:1–19. doi: 10.3390/cancers13163968 PMC8394398 34439120

[38] Mexican Social Security Institute (IMSS) Mexican Government. Clinical practice guidelines. Treatment of cervical cancer at the second and third-level of care (2017). Available online at: https://www.imss.gob.mx/sites/all/statics/guiasclinicas/333GER.pdf (Accessed June 29, 2024).

[39] Hajian-Tilaki K. Sample size estimation in diagnostic test studies of biomedical informatics. J BioMed Inform. (2014) 48:193–204. doi: 10.1016/j.jbi.2014.02.013 24582925

[40] Makde MM Sathawane P. Liquid-based cytology: Technical aspects. CytoJournal. (2022) 19(41):1–13. doi: 10.25259/CMAS_03_16_2021 PMC9345114 35928530

[41] Strander B Andersson-Ellström A Milsom I Rådberg T Ryd W. Liquid-based cytology versus conventional Papanicolaou smear in an organized screening program : a prospective randomized study. Cancer. (2007) 111(5):285–91. doi: 10.1002/cncr.22953 17724676

[42] Cheung ANY Szeto EF Leung BSY Khoo US Ng AWY. Liquid-based cytology and conventional cervical smears: A comparison study in an Asian screening population. Cancer. (2003) 99(6):331–5. doi: 10.1002/cncr.11786 14681939

[43] Grupo Diagnóstico Aries. Laboratorios Dr. Moreira (2023). Available online at: https://www.labmoreira.com/ (Accessed June 29, 2024).

[44] Hospital Ginequito. Centro Médico de la Mujer (2023). Available online at: https://www.ginequito.com.mx/ (Accessed June 29, 2024).

[45] DIPAC Lab. DIPAC Laboratory. Pathological Anatomy and Cytopathology (2022). Available online at: https://www.dipac-lab.net/ (Accessed June 29, 2024).

[46] Innbiogem SC. Vitagenesis Lab (2024). Available online at: https://vitagenesis.odoo.com/en/home (Accessed June 29, 2024).

[47] Mexican Postal Service (SEPOMEX) Mexican Government. Postal Code Online Search (2022). Available online at: https://www.correosdeMexico.gob.mx/SSLServicios/ConsultaCP/Descarga.aspx (Accessed June 29, 2024).

[48] National Council for Evaluation of Social Development Policy (CONEVAL) Mexican Government. Poverty Assessment. Poverty-level by urban locality (2022). Available online at: https://www.coneval.org.mx/Medicion/Paginas/pobreza_localidad_urbana.aspx (Accessed June 29, 2024).

[49] R Core Team. R: A Language and Environment for Statistical Computing. Viena: R Foundation for Statistical Computing (2023). Available at: https://www.R-project.org.

[50] Wickham H. ggplot2: Elegant Graphics for Data Analysis. New York: Springer-Verlag (2016). Available at: https://ggplot2.tidyverse.org.

[51] Trevethan R. Sensitivity, specificity, and predictive values: foundations, pliabilities, and pitfalls in research and practice. Front Public Health. (2017) 5. doi: 10.3389/fpubh.2017.00307 PMC5701930 29209603

[52] Parikh R Mathai A Parikh S Chandra Sekhar G Thomas R. Understanding and using sensitivity, specificity and predictive values. Indian J Ophthalmol. (2008) 56(1):45–50. doi: 10.4103/0301-4738.37595 PMC2636062 18158403

[53] McNamara LA Martin SW. Principles of Epidemiology and Public Health. In: Principles and Practice of Pediatric Infectious Diseases. Philadelphia, PA, US: Elsevier Inc (2018). p. 1–9.e1.

[54] Robin X Turck N Hainard A Tiberti N Lisacek F Sanchez JC. pROC: An open-source package for R and S+ to analyze and compare ROC curves. BMC Bioinf. (2011) 12(77):1–8. doi: 10.1186/1471-2105-12-77 PMC3068975 21414208

[55] Revelle W. psych: Procedures for Psychological, Psychometric, and Personality Research. Evanston, Illinois: Northwestern University (2024). Available at: https://CRAN.R-project.org/package=psych.

[56] Landis JR Koch GG. The measurement of observer agreement for categorical data. Biometrics. (1977) 33:159–74. doi: 10.2307/2529310 843571

[57] Farzanehpour M Muhammadnejad A Akhavan S Nader A Razavi E Jalilvand S. P16INK4A immunohistochemistry as a gold standard for cervical cancer and precursor lesions screening. Iran J Public Health. (2020) 49:312–22. http://ijph.tums.ac.ir. PMC7231710 32461939

[58] Silva DC Gonçalves AK Cobucci RN Mendonça RC Lima PH Cavalcanti G. Immunohistochemical expression of p16, Ki-67 and p53 in cervical lesions – A systematic review. Pathol Res Pract. (2017) 213:723–9. doi: 10.1016/j.prp.2017.03.003 28554769

[59] Lin J Albers AE Qin J Kaufmann AM. Prognostic significance of overexpressed p16INK4ain patients with cervical cancer: A meta-analysis. PloS One. (2014) 9(9):e106384. doi: 10.1371/journal.pone.0106384 25188353 PMC4154680

[60] Tsoumpou I Arbyn M Kyrgiou M Wentzensen N Koliopoulos G Martin-Hirsch P. p16INK4a immunostaining in cytological and histological specimens from the uterine cervix: A systematic review and meta-analysis. Cancer Treat Rev. (2009) 35:210–20. doi: 10.1016/j.ctrv.2008.10.005 PMC2784486 19261387

[61] Wilson JMG Jungner G. Principles and practice of screening for disease. Geneva: World Health Organization (1968) pp. 1–168.

[62] Cheung TH Yim SF Yu MY Worley MJ Fiascone SJ Chiu RWK. Liquid biopsy of HPV DNA in cervical cancer. J Clin Virol. (2019) 114:32–6. doi: 10.1016/j.jcv.2019.03.005 30913520

[63] Thangarajah F Busshoff J Salamon J Pruss MS Lenz C Morgenstern B. Digital droplet PCR-based quantification of ccfHPV-DNA as liquid biopsy in HPV-driven cervical and vulvar cancer. J Cancer Res Clin Oncol. (2023) 149:12597–604. doi: 10.1007/s00432-023-05077-3 PMC10587338 37452202

[64] Leffers M Herbst J Kropidlowski J Prieske K Bohnen AL Peine S. Combined liquid biopsy methylation analysis of CADM1 and MAL in cervical cancer patients. Cancers (Basel). (2022) 14(16):1–11. doi: 10.3390/cancers14163954 PMC9406083 36010947

[65] Du S Zhao Y Lv C Wei M Gao Z Meng X. Applying serum proteins and microRNA as novel biomarkers for early-stage cervical cancer detection. Sci Rep. (2020) 10(1):1–8. doi: 10.1038/s41598-020-65850-z PMC7271168 32493989

[66] Zhu X Jin C Pan Q Hu X. Determining the quantitative relationship between glycolysis and GAPDH in cancer cells exhibiting the Warburg effect. J Biol Chem. (2021) 296(100369):1–20. doi: 10.1016/j.jbc.2021.100369 PMC7960551 33545174

[67] Loh TJ Moon H Cho S Jang H Liu YC Tai H. CD44 alternative splicing and hnRNP A1 expression are associated with the metastasis of breast cancer. Oncol Rep. (2015) 34:1231–8. doi: 10.3892/or.2015.4110 26151392

[68] International Agency for Research on Cancer. CanScreen5. Cervical cancer screening programme. Country fact sheet: Mexico. Lyon: World Health Organization (2021). Available at: https://canscreen5.iarc.fr/?page=countryfactsheetcervix&q=MEX&rc.

[69] Chamber of Deputies. H. Congress of the Union. Regulations of the General Health Law on Research for Health. Mexico City, Mexico (2014). pp. 1–31.

