# Supplementary material for: Pioneering molecular screening for cervical precursor lesions and cervical cancer in sera
Source: Front Oncol. 2024 Nov 14;14:1483882. doi: 10.3389/fonc.2024.1483882 (PMC11602649; doi:10.3389/fonc.2024.1483882)
Supplement: Supplementary file 1 [file DataSheet1.docx]

Supplementary Material

# Supplementary Figures and Tables

Paper: Pioneering molecular screening for cervical precursor lesions and cervical cancer in sera.

## Supplementary Figures

**
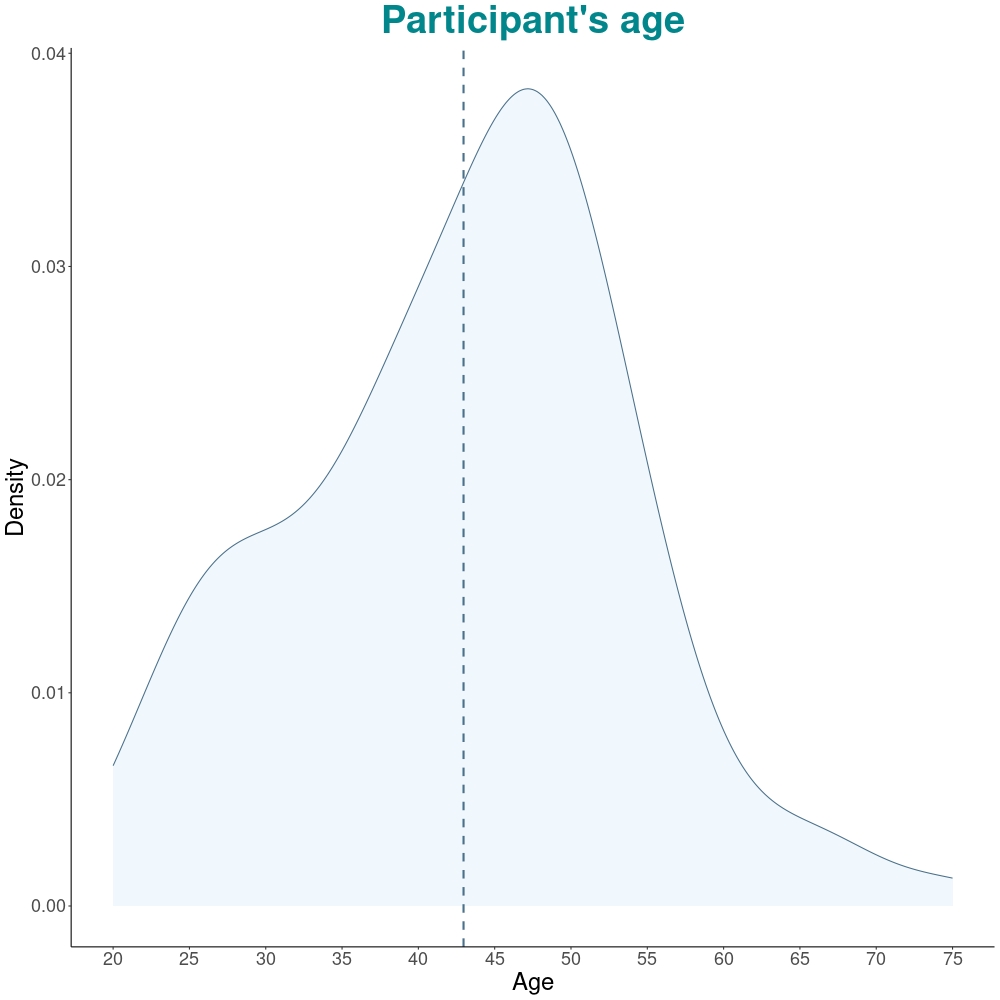
**

**Supplementary Figure 1.** Age of participants. The dotted line represents the mean age (42.96 years). Data from 99 participants.


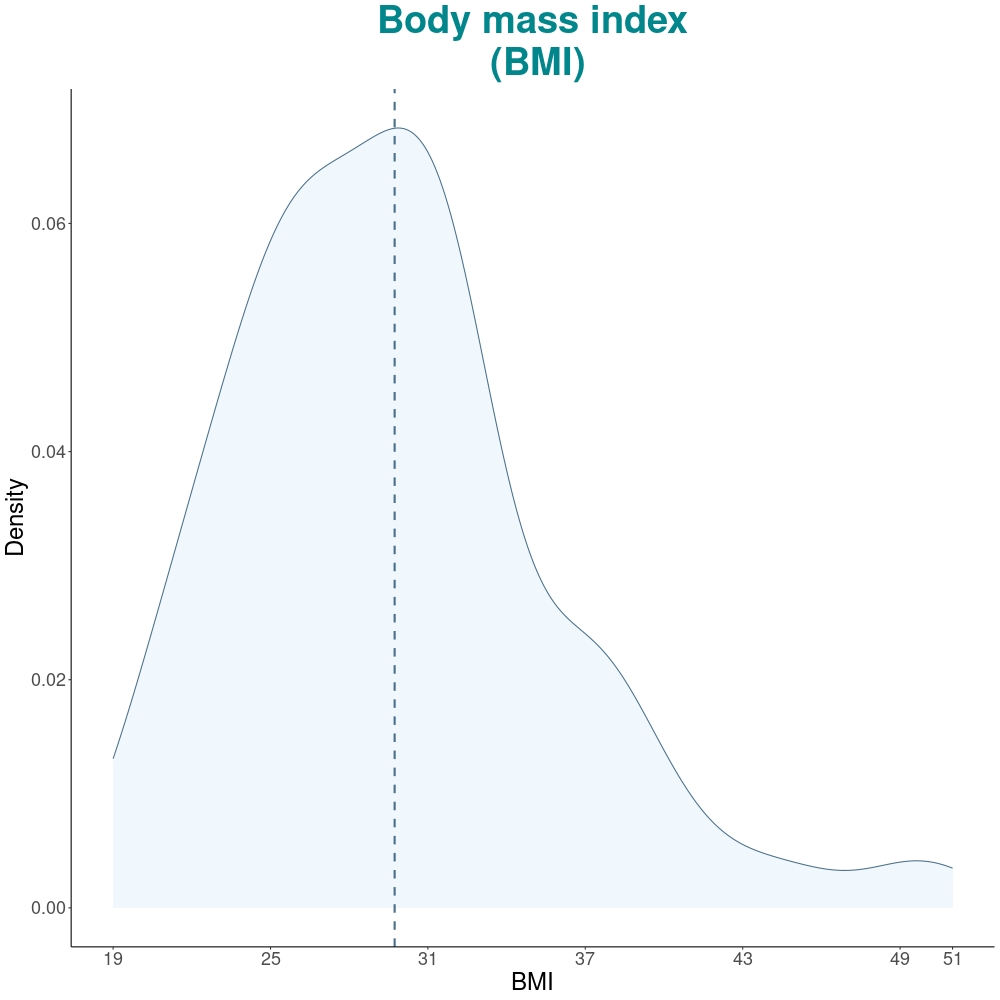


**Supplementary Figure 2.** Body mass index (BMI). The dotted line denotes mean BMI (29.73 kg/m^2^). Data from 94 participants.


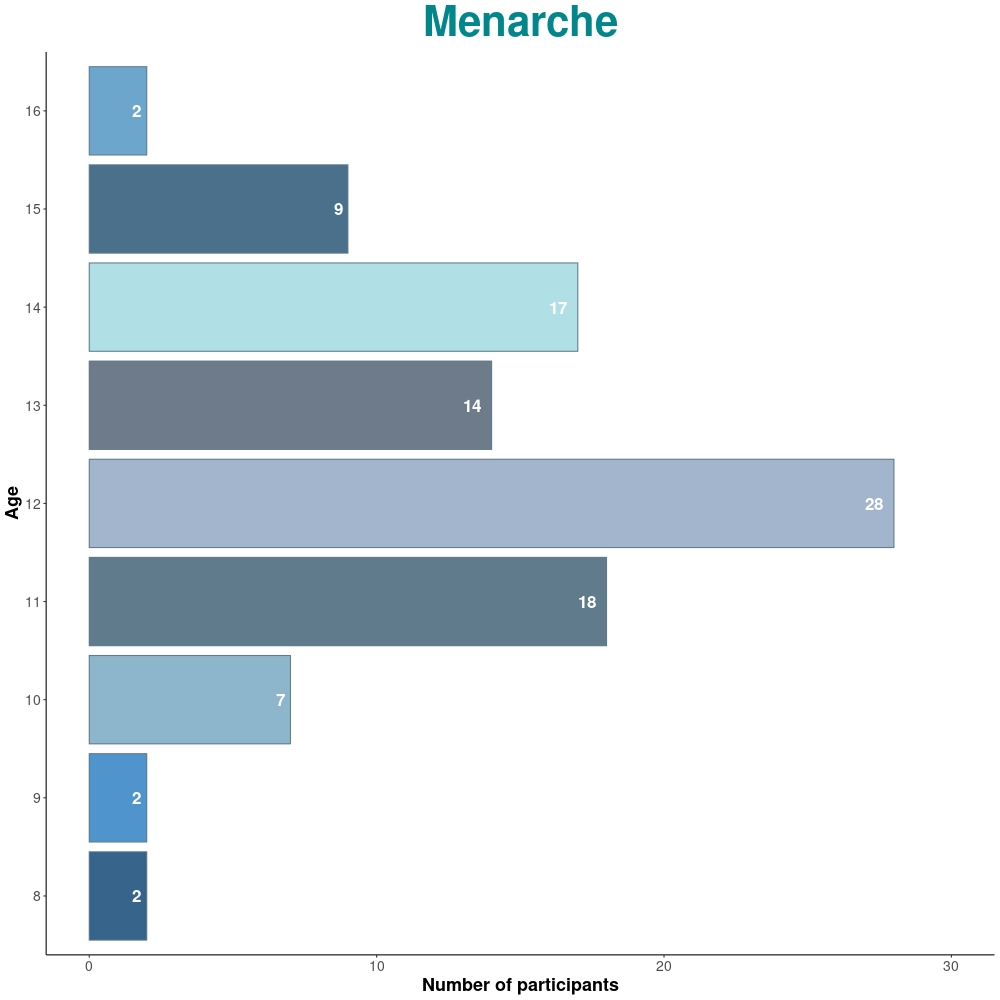


**Supplementary Figure 3.** Participant’s age of menarche. The mean age of menarche was 12.37 years. Numbers inside bars indicate the total number of participants per category. Data from 99 participants.


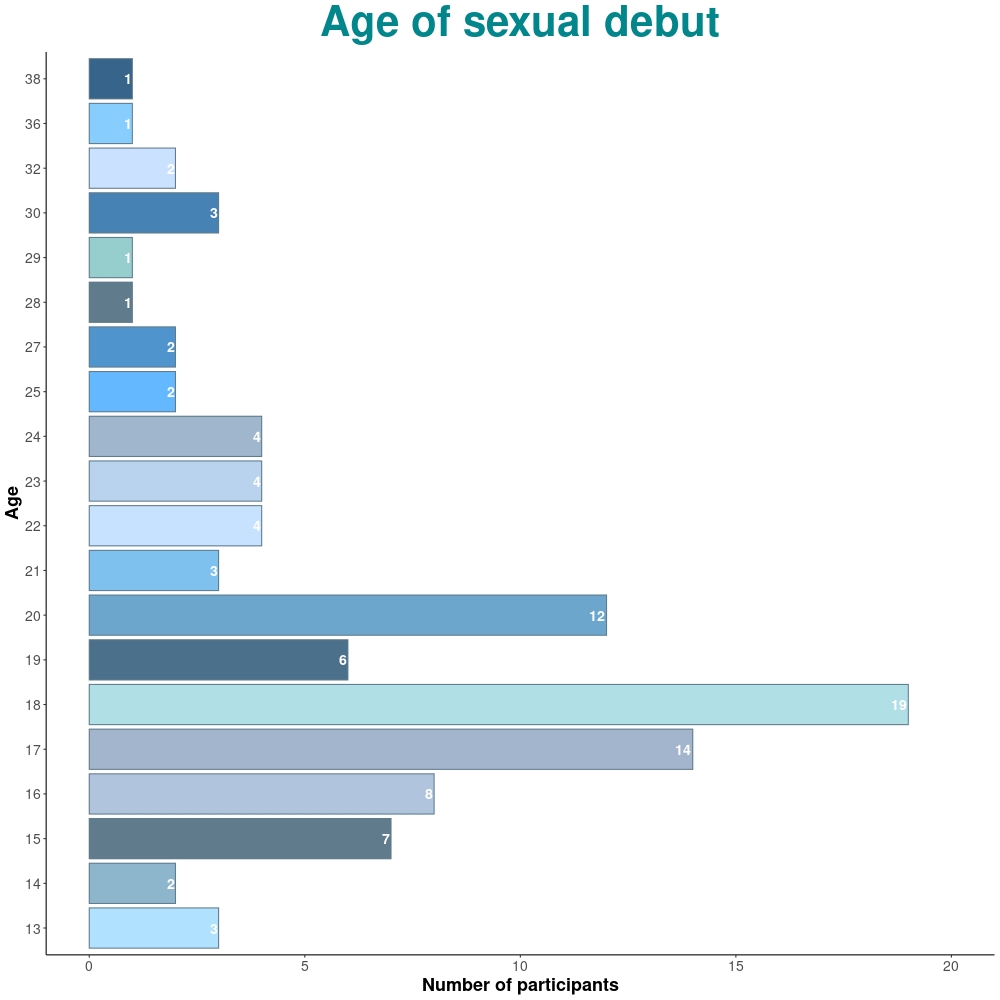


**Supplementary Figure 4.** Age of sexual debut. Numbers inside bars indicate the total number of participants per category. The mean age of sexual debut was 19.82 years. Data from 99 participants.


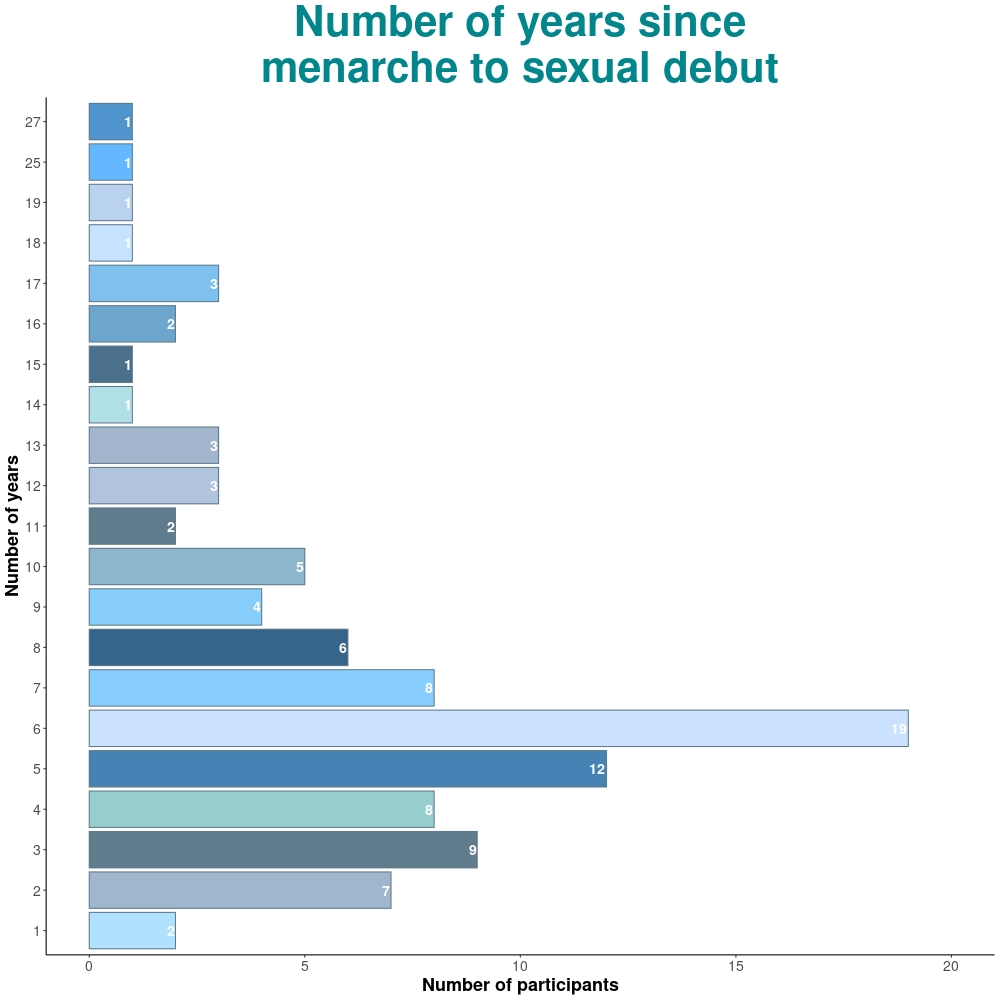


**Supplementary Figure 5.** Number of years since menarche to sexual debut, calculated by subtracting the age of menarche from the age of sexual debut. Numbers inside bars denote the total number of participants per category. The mean number of years since menarche to sexual debut was 7.44 years. Data from 99 participants.


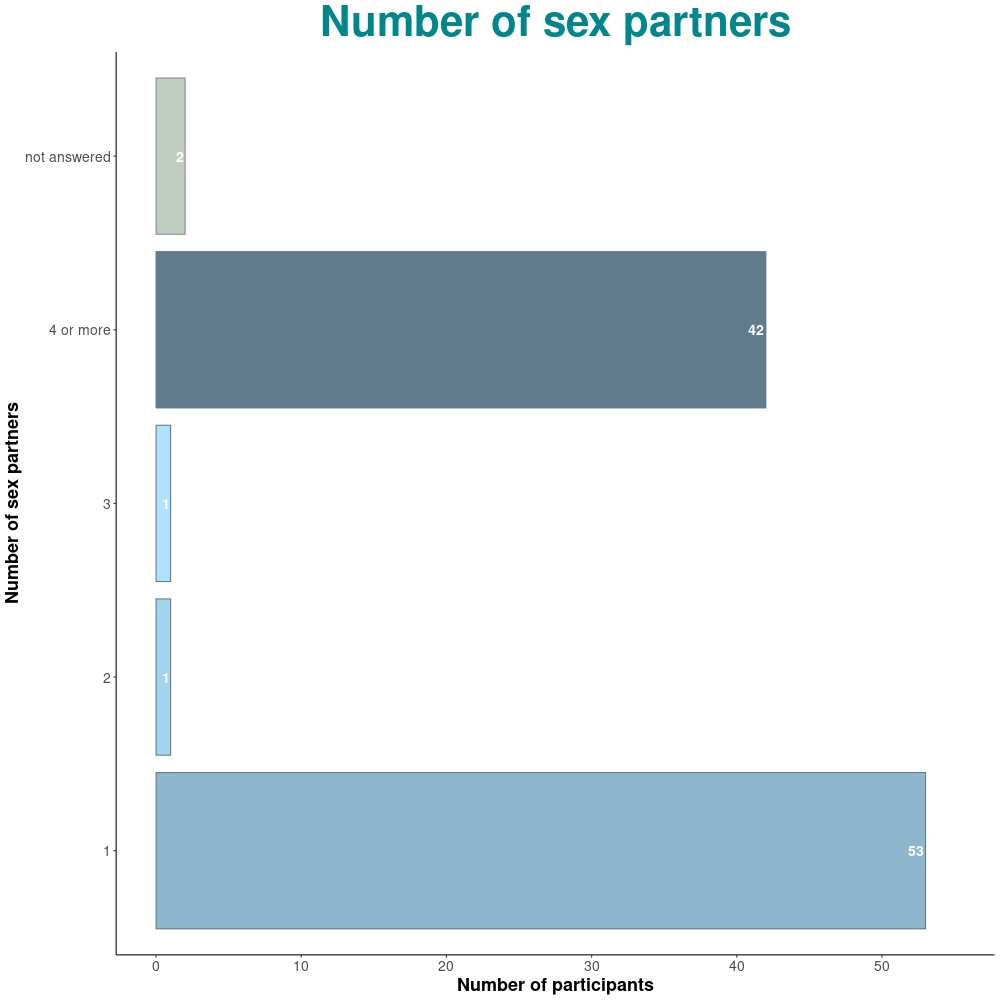


**Supplementary Figure 6.** Number of lifetime sexual partners. Numbers inside bars denote the total number of participants per category. Data from 99 participants, where two of them did not answer this question.


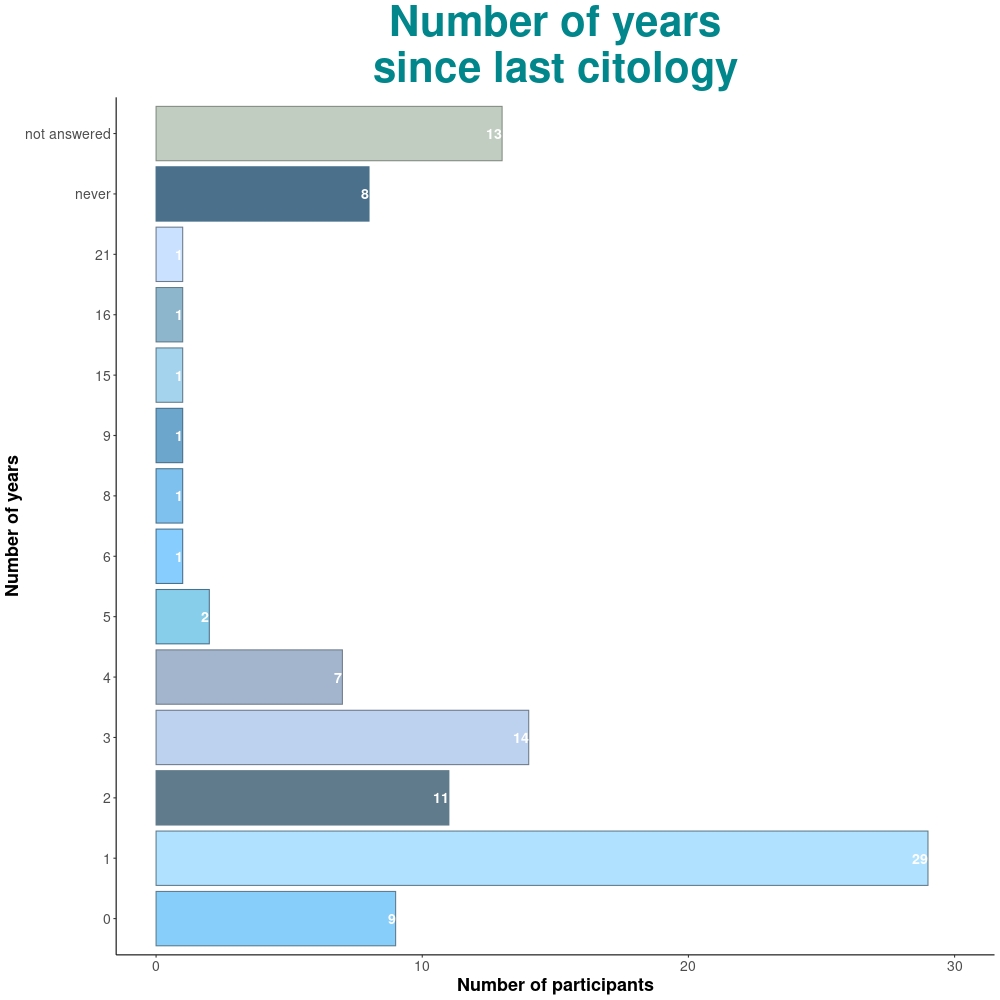


**Supplementary Figure 7.** Number of years since participant received her last cytology. Numbers inside bars denote the total number of participants per category. Data from 99 participants.


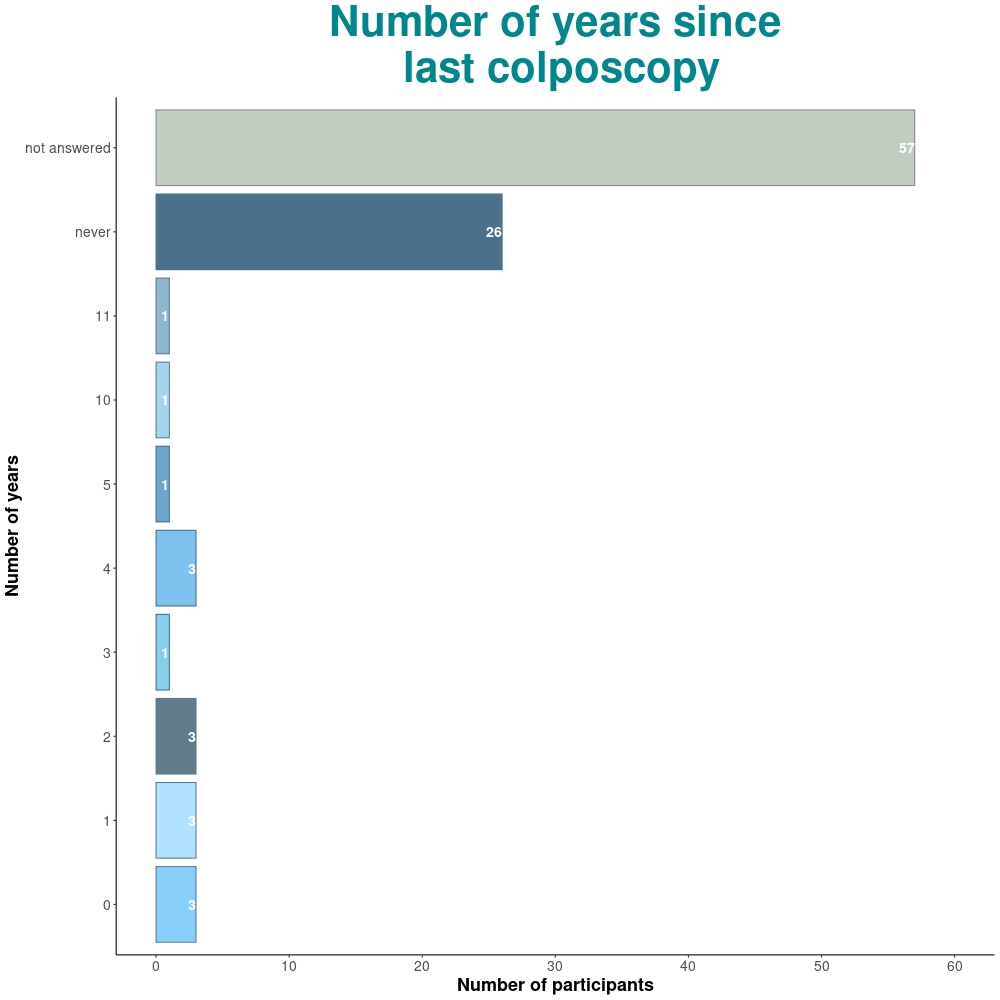


**Supplementary Figure 8.** Number of years since participant received her last colposcopy. Numbers inside bars denote the total number of participants per category. Data from 99 participants.


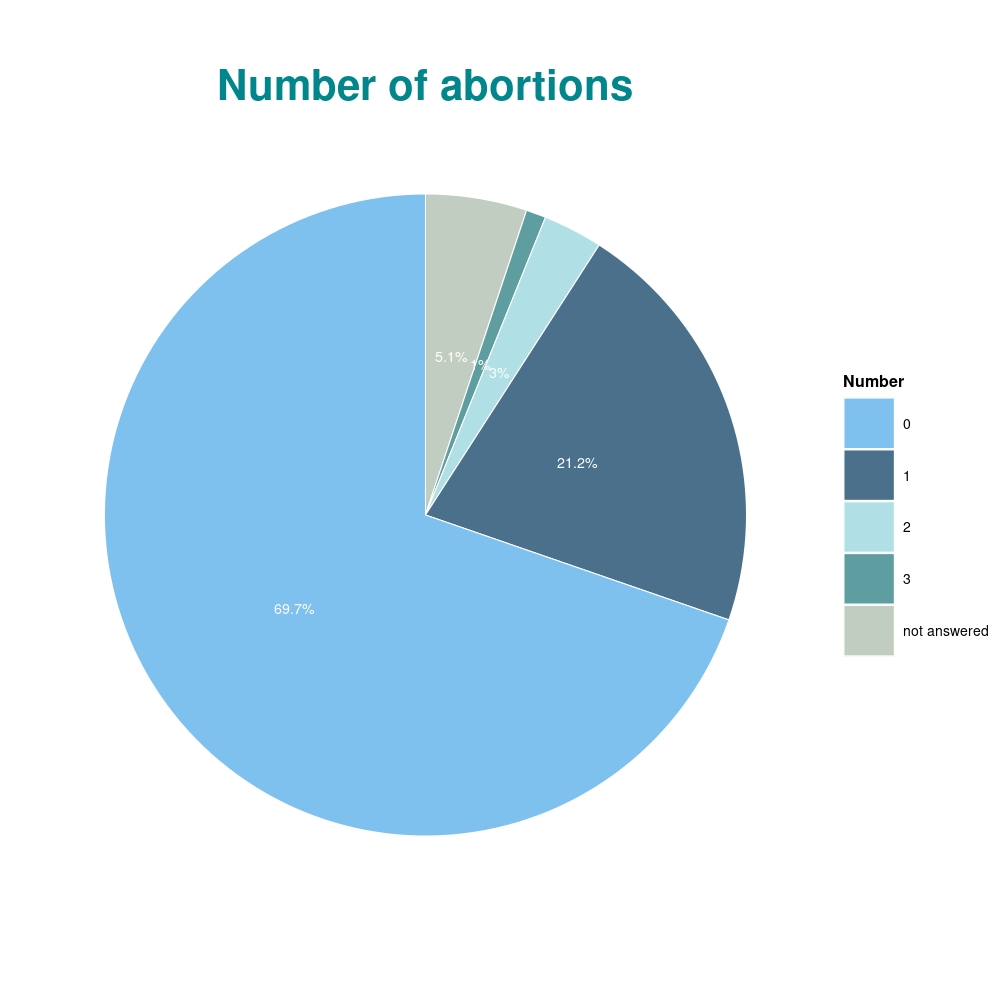


**Supplementary Figure 9.** Number of abortions. Numbers inside sections denote the percentages of participants per category. Data from 99 participants, where five of them did not answer this question.


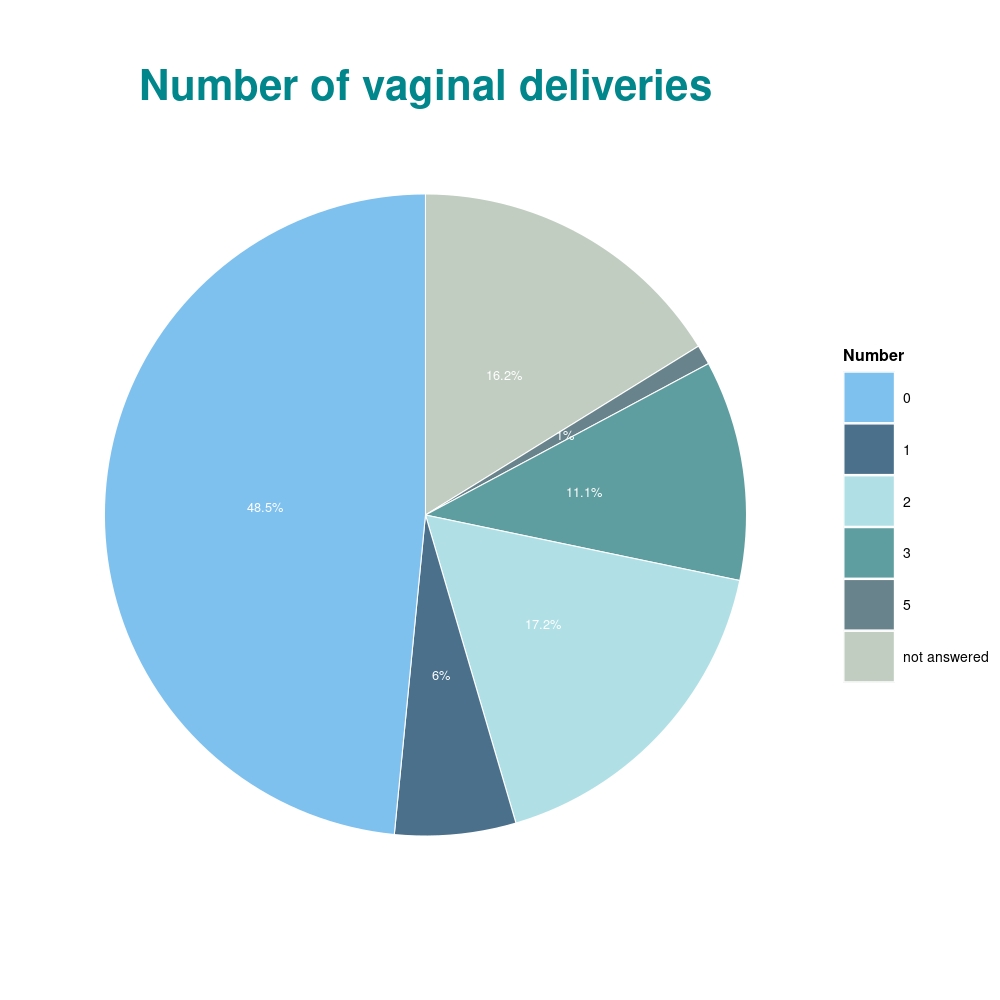


**Supplementary Figure 10.** Number of vaginal deliveries. Numbers inside sections denote the percentages of participants per category. Data from 99 participants, where 16 of them did not answer this question.


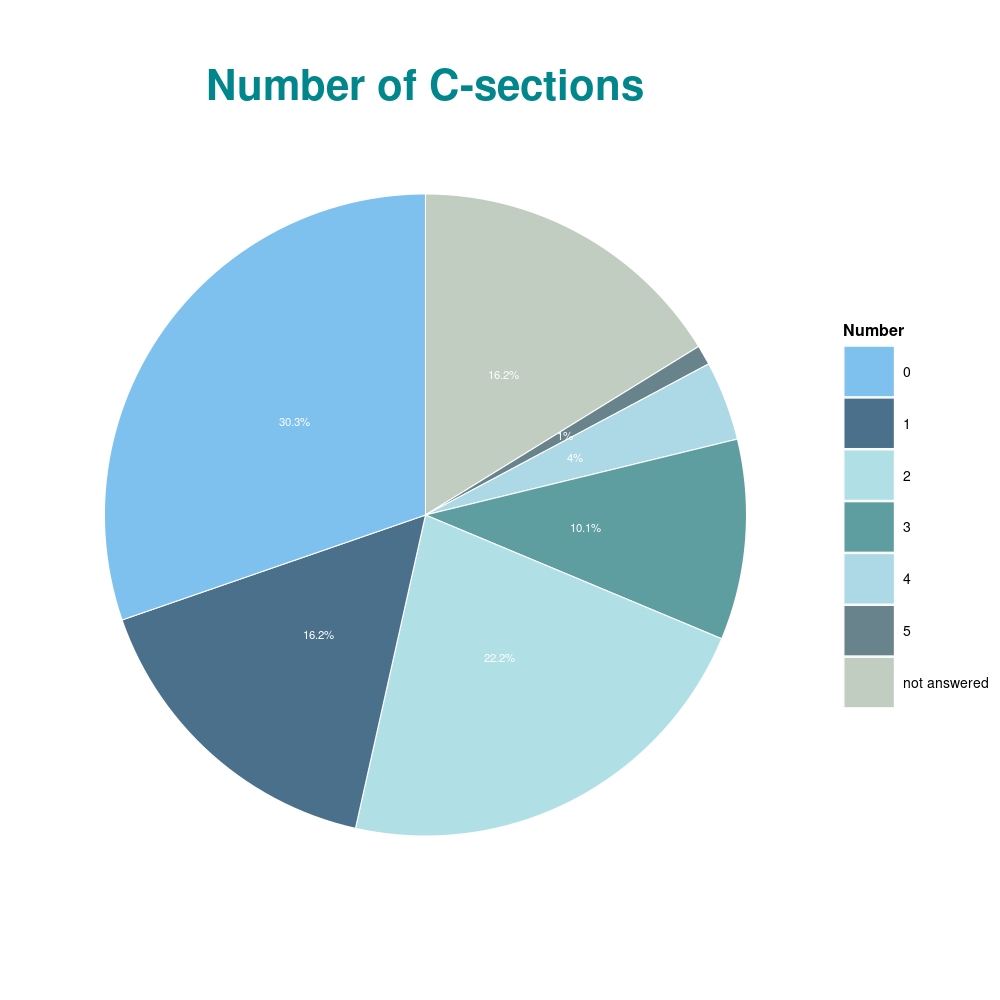


**Supplementary Figure 11.** Number of Cesarean sections. Numbers inside sections denote the percentages of participants per category. Data from 99 participants, where 16 of them did not answer this question.


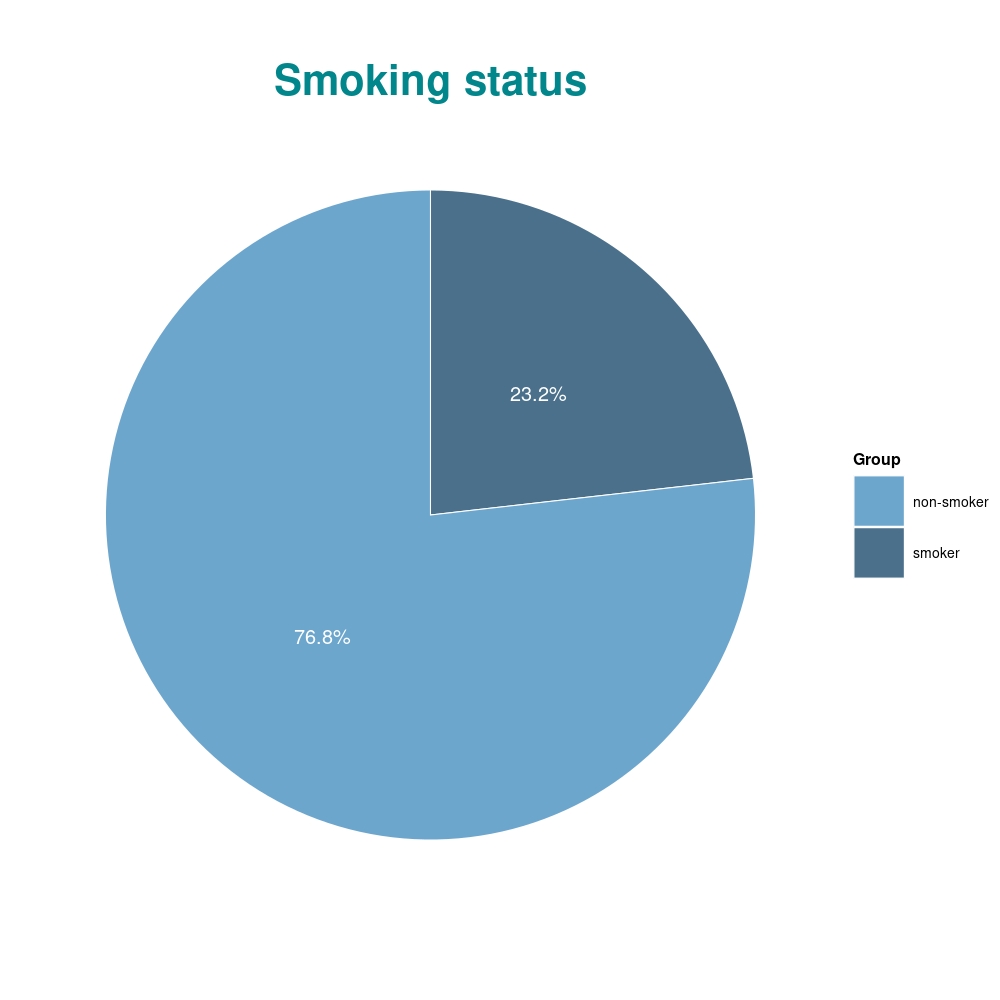


**Supplementary Figure 12.** Smoking status. The smoker category includes quitters and persistent smokers. Numbers inside sections denote the percentages of participants per category. Data from 99 participants.


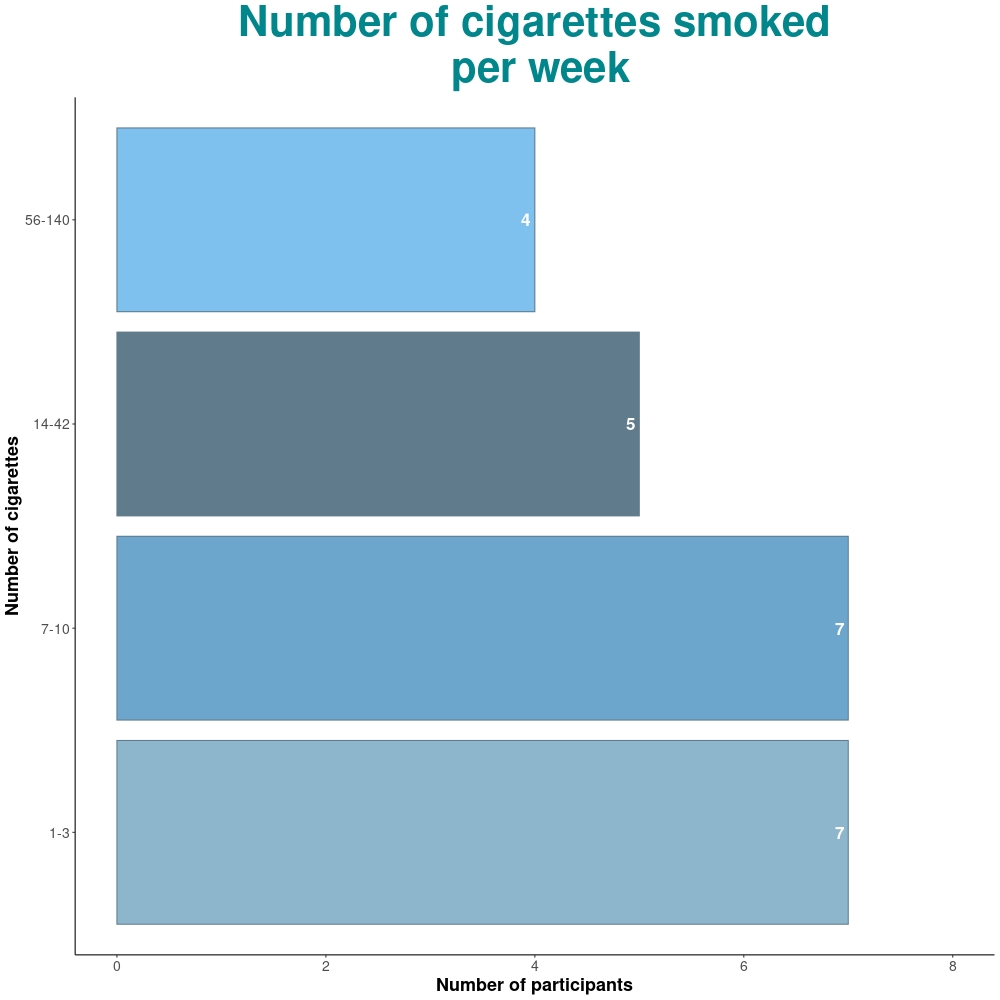


**Supplementary Figure 13.** Number of cigarettes per week. Numbers inside bars denote the total number of participants per rank. Data from 23 participants.


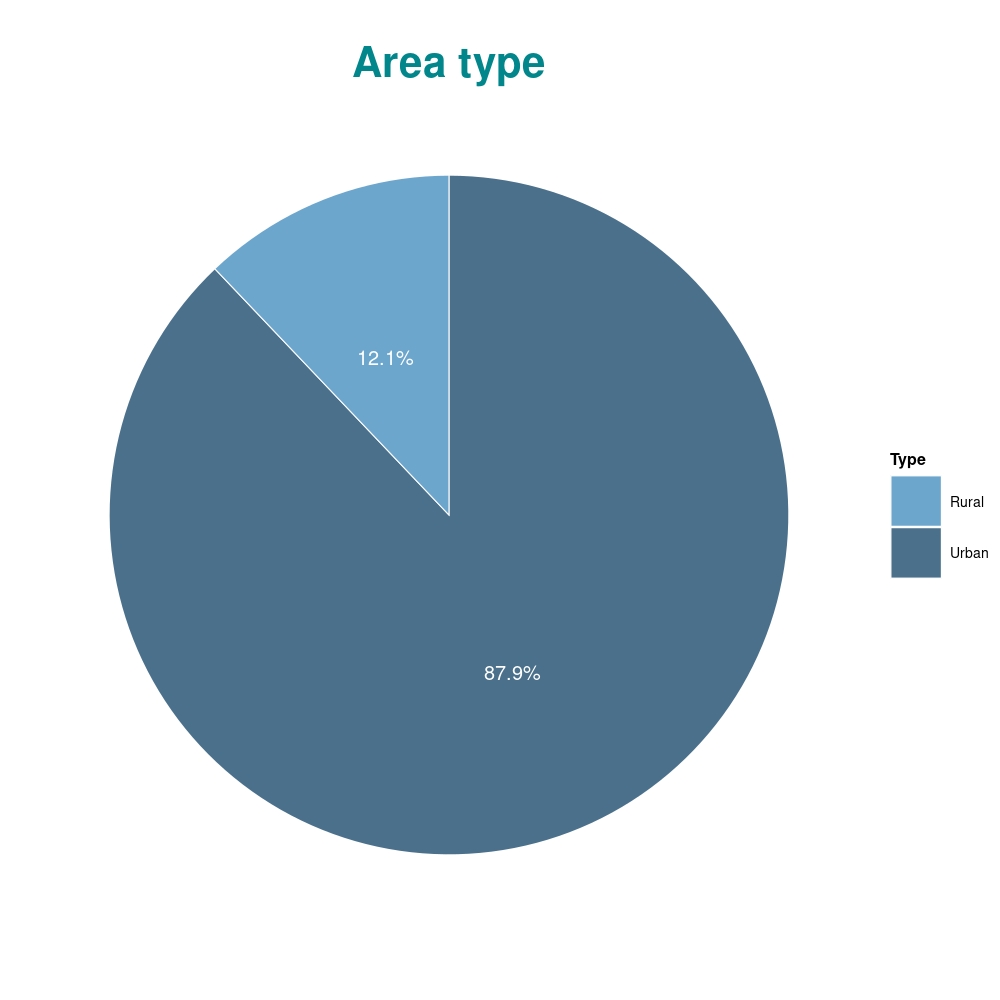


**Supplementary Figure 14.** Area of residence. Numbers inside sections denote the percentages of participants per area type. Data from 99 participants. Area type was determined based on postal code and/or full address of participants according to the Mexican Post Service (SEPOMEX) classification.


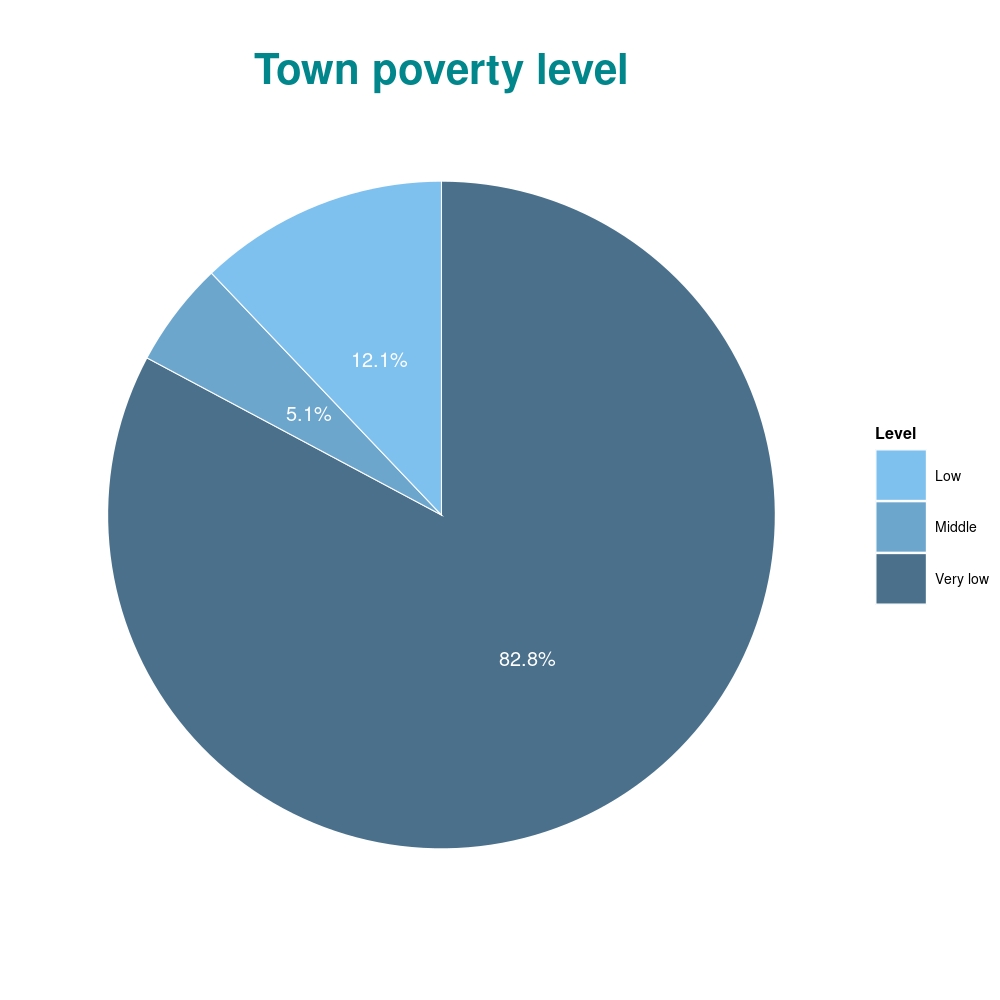


**Supplementary Figure 15.** Town poverty level. Numbers inside sections denote the percentages of participants per level. Data from 99 participants. The postal code of participant was used to determine the town poverty level according to the National Council for Evaluation of Social Development Policy (CONEVAL) classification.

## Supplementary Tables

**Supplementary Table 1.** Demographic and clinical data of all participants (*Table-S1.xlsx*).
